# Comparative efficacy and safety of PCSK9 inhibitors for lipoprotein(a) reduction: a systematic review and network meta−analysis

**DOI:** 10.1186/s12872-026-06480-7

**Published:** 2026-08-18

**Authors:** Ying Pu, Wenrui Huang, Yan Tu, Qingchun Zeng

**Affiliations:** 1 Department of Cardiology, State Key Laboratory of Multi−Organ Injury Prevention and Treatment, National Clinical Research Center for Kidney and Urological Diseases, Nanfang Hospital, Southern Medical University, Guangzhou, 510515 China; 2 Shenzhen Traditional Chinese Medicine Hospital, Shenzhen, Guangdong China

**Keywords:** Proprotein convertase subtilisin/kexin type 9 inhibitors, Hypercholesterolemia, Dyslipidemia, Lipoprotein(a), Network meta−analysis

## Abstract

**Introduction:**

Lipoprotein(a) [Lp(a)] is a genetically determined cardiovascular risk factor that is largely unaffected by statins. Although PCSK9 inhibitors can lower Lp(a), comparative evidence among agents is limited.

**Methods:**

We conducted a systematic review and frequentist random−effects network meta−analysis of randomized controlled trials from database inception to February 2026 (PROSPERO: CRD420251208404). The primary outcome was the percentage change in Lp(a) from baseline. Secondary outcomes included changes in low−density lipoprotein cholesterol (LDL − C), non−high−density lipoprotein cholesterol (non − HDL−C), triglyceride (TG), total cholesterol (TC), high−density lipoprotein cholesterol (HDL − C), and apolipoprotein B (ApoB). Safety outcomes were adverse events (AEs) and serious adverse events (SAEs). Certainty of evidence was assessed using CINeMA. Sensitivity analyses were conducted according to the reported Lp(a) unit and by restricting the analysis to label−approved dose regimens.

**Results:**

Sixty − two trials involving 73,240 participants and six PCSK9 inhibitors were included. All evaluated agents were associated with reductions in Lp(a) versus placebo. The largest point estimates were observed for ongericimab (MD = − 46.36%, 95% CI − 55.96 to − 36.77) and tafolecimab (MD = − 41.12%, 95% CI − 50.73 to − 31.51), although certainty was low and comparisons were predominantly indirect. Sensitivity analyses yielded broadly similar results. No statistically significant differences in overall AEs or SAEs were detected versus placebo.

**Conclusions:**

PCSK9 inhibitors may reduce Lp(a) and improve atherogenic lipid profiles. Comparative effects among individual agents remain uncertain because the evidence was mainly indirect and of low certainty. Whether Lp(a) lowering itself improves cardiovascular outcomes remains unknown.

**Supplementary Information:**

The online version contains supplementary material available at https://doi.org/10.1186/s12872-026-06480-7.

## Introduction

Atherosclerotic cardiovascular disease (ASCVD) remains the primary cause of mortality and disability worldwide, exerting a persistent and profound impact on public health systems [1]. Statin−based lipid−lowering therapy has long been proven effective in reducing low−density lipoprotein cholesterol (LDL − C) and serves as the cornerstone for decreasing cardiovascular events [2]. However, even among patients who achieve recommended LDL − C targets, a substantial proportion of individuals still experience atherosclerotic events [3]. This “residual cardiovascular risk” suggests that lipid components other than LDL − C play an independent and critical role in the pathogenesis of ASCVD, while multiple studies have indicated a potential causal association between elevated Lipoprotein(a) [Lp(a)] and ASCVD risk [4, 5].

Lp(a) is a genetically regulated lipoprotein that contributes to atherosclerosis and thrombosis, independent of traditional risk factors [6, 7]. Structurally, Lp(a) is composed of an LDL−like core covalently bonded to apolipoprotein(a) [Apo(a)], which possesses variable copy numbers; this configuration confers complex biological properties, including pro−inflammatory, pro−oxidative, and anti−fibrinolytic characteristics [8]. Elevated Lp(a) levels are strongly associated with the risks of coronary atherosclerosis, aortic valve calcification, and ischemic vascular events [9, 10]. Notably, commonly prescribed statins exert limited effects on Lp(a) levels and may even result in a slight elevation, further underscoring the status of Lp(a) as a core component of residual risk following statin therapy [6, 7, 11]. However, the precise quantitative contribution of this residual risk warrants further refined investigation.

The introduction of proprotein convertase subtilisin/kexin type 9 (PCSK9) inhibitors has established a novel therapeutic avenue for the management of hypercholesterolemia and dyslipidemia. By inhibiting the degradation of low−density lipoprotein receptors (LDLR), these agents substantially upregulate the expression and recycling of hepatic surface receptors, thereby accelerating LDL − C clearance [12, 13]. However, the underlying molecular mechanisms remain a subject of intensive investigation and considerable debate within the field of lipidology, with a definitive consensus yet to be reached [14]. It has been hypothesized that increased LDLR availability may enhance clearance of Lp(a), which contains an LDL−like core, by reducing competitive inhibition from LDL [12, 14]. Some studies also suggest that PCSK9 inhibitors could influence Apo(a) synthesis or hepatic assembly of Lp(a) particles [15, 16]; however, these mechanisms remain to be fully validated.

PCSK9 inhibitors differ substantially in pharmacokinetics and lipid−lowering potency. Monoclonal antibodies (mAbs), such as evolocumab and alirocumab, require subcutaneous administration every two weeks or monthly to maintain stable effects [17]. Inclisiran, a small interfering RNA (siRNA) agent, inhibits hepatic PCSK9 synthesis and can be administered once every six months after initial loading doses [18]. Overall, PCSK9 inhibitors reduce Lp(a) by approximately 20–30% [19, 20], although the magnitude varies across agents and populations, highlighting opportunities for optimizing novel therapeutics. In certain studies, the reduction associated with evolocumab approached or exceeded 30% [21], whereas the mean reduction for alirocumab in several trials was between 15% and 20% [14, 20]. Although novel agents such as tafolecimab, recaticimab, and ongericimab have entered clinical application, direct head − to−head randomized controlled trial (RCT) data comparing these new drugs with conventional mAbs or siRNA are currently unavailable. To address this gap, we conducted this systematic review and network meta−analysis. By synthesizing direct and indirect evidence, this study aims to quantify and compare the relative efficacy of these agents in modulating Lp(a) and other lipid indicators, providing an evidence−based reference for the precision management of individuals with dyslipidemia.

## Methods

### Protocol and registration

This systematic review and network meta−analysis (NMA) was designed and reported in strict accordance with the Preferred Reporting Items for Systematic Reviews and Meta−Analyses (PRISMA) 2020 statement and its extension for network meta−analyses (PRISMA − NMA) [22, 23]. The study protocol was prospectively registered with PROSPERO (Registration No.: CRD420251208404). All methodological procedures were conducted following the principles established in the Cochrane Handbook for Systematic Reviews of Interventions to ensure the highest degree of transparency and scientific rigor.

### Search strategy and information sources

A comprehensive and systematic literature search was conducted across PubMed, Web of Science, the Cochrane Central Register of Controlled Trials (CENTRAL) and Embase from database inception to February 2026, without language restrictions. The search utilized a combination of Medical Subject Headings (MeSH) terms and free−text keywords specifically targeting various PCSK9 inhibitors, including established agents such as evolocumab, alirocumab, and inclisiran, as well as novel therapeutics including tafolecimab, recaticimab, and ongericimab. The specific search strings employed are detailed in Appendix 1. Furthermore, we manually screened ClinicalTrials.gov, conference proceedings (ESC, ACC, and AHA), and reference lists of identified studies to capture gray literature and additional eligible trials.

### Eligibility criteria

The inclusion and exclusion criteria for this systematic review and network meta−analysis were predefined according to the PICOS (Population, Intervention, Comparator, Outcome, and Study design) framework.

### Types of participants

Eligible studies included adults (age ≥ 18 years) diagnosed with hypercholesterolemia or dyslipidemia, regardless of whether they had established ASCVD. No specific baseline threshold for Lp(a) was required for study enrollment.

### Types of interventions and comparators

Interventions comprised any PCSK9 inhibitors within clinical dosage ranges, including both mAbs (alirocumab, evolocumab, tafolecimab, recaticimab and ongericimab) and siRNA (inclisiran). These agents could be administered as either monotherapy or in combination with background lipid−lowering therapies. Eligible comparators consisted of placebo or other active PCSK9 inhibitors.

### Types of outcomes

The primary outcome was the percentage change in Lp(a) from baseline at the prespecified primary endpoint of each trial. Secondary outcomes were percentage changes in LDL − C, non−high−density lipoprotein cholesterol (non − HDL−C), triglyceride (TG), total cholesterol (TC), high−density lipoprotein cholesterol (HDL − C) and apolipoprotein B (ApoB). Safety outcomes were assessed separately as adverse events (AEs) and serious adverse events (SAEs). Specific AEs, including injection−site reactions, muscle−related events, hepatic effects, neurocognitive events, diabetes or glucose−control events, nasopharyngitis, upper respiratory tract infection, urinary tract infection, and headache, were also extracted when reported.

### Study design and selection

Only RCTs with a treatment duration of at least 8 weeks were included. Studies were excluded if they met the following criteria: (1) non−randomized studies (e.g., observational or cohort studies); (2) duplicate publications or reports with overlapping patient populations; (3) trials with insufficient data for the outcomes of interest.

### Data extraction

Data extraction was performed independently by two investigators (YP and WYH) with a third senior reviewer acting as an adjudicator for any discrepancies (QCZ). Extracted information included study characteristics, participant demographics, dose regimens, follow − up duration, and intervention details. For specific AEs, event counts, percentages, and definitions were extracted as reported in the original trials. For data presented exclusively in graphical format, numerical values were estimated using WebPlot Digitizer version 5.2 [24].

### Quality assessment

The risk of bias for each included RCT was appraised using the Cochrane Risk of Bias tool (RoB 2.0) across five domains: randomization process, deviations from intended interventions, missing outcome data, measurement of the outcome, and selection of the reported result [25]. Publication bias and small−study effects were scrutinized using comparison−adjusted funnel plots, which are specifically designed to account for the multiple treatment comparisons in a network structure. Finally, the certainty and quality of the evidence for each network estimate were appraised using the Confidence in Network Meta−Analysis (CINeMA) framework across six domains: within−study bias, reporting bias, indirectness, imprecision, heterogeneity, and inconsistency [26, 27].

### Data transformation

To address differences in reported units, all lipid data were synthesized as percentage changes from baseline. When the standard deviation (SD) was not reported, it was derived from the 95% confidence interval (CI) or standard error (SE) using Cochrane−recommended formulae ($SE=\frac{\text{Upper Limit}-\text{Lower Limit}}{3.92}$; $SD=\mathrm{SE}\:\times\:\sqrt{\mathrm{n}}$) [28]. For trials reporting medians and interquartile ranges (IQRs), we used estimation approaches according to distributional assumptions. The Quantile Estimation (QE) or Box−Cox (BC) methods proposed by McGrath et al. [29] were prioritized for skewed variables, particularly Lp(a), whereas the method of Wan et al. [30] was applied to approximately normally distributed lipid variables. When variances remained unavailable, they were imputed from other arms within the same trial or from clinically similar trials in the network.

### Statistical analysis

Network meta−analysis was performed using Stata version 18.0 within a frequentist framework [31]. We employed a multivariate random−effects model estimated via the restricted maximum likelihood method to synthesize direct and indirect evidence [32]. Continuous outcomes were expressed as mean differences (MDs) with 95% CIs, while binary safety outcomes were calculated as odds ratios (ORs). Between−study heterogeneity was quantified using the τ^2^ statistic, with values of 0.04, 0.16, and 0.36 representing low, moderate, and high heterogeneity, respectively [33]. We evaluated transitivity by comparing age, baseline Lp(a), and baseline LDL − C across treatment nodes. To explore potential sources of heterogeneity, subgroup analyses for the primary outcome were planned, stratified by follow − up duration, background statin therapy, and geographical region. To assess the robustness of the primary findings, sensitivity analyses were conducted according to the reported Lp(a) unit (mg/dL vs. nmol/L) and by restricting the network to label−approved dose regimens. Specific adverse events were summarized descriptively because their definitions, ascertainment methods, and reporting completeness varied across trials; therefore, these outcomes were not quantitatively pooled. Treatment hierarchies were established using the surface under the cumulative ranking (SUCRA) curve [34].

## Results

### Literature selection and study characteristics

**Fig. 1:**
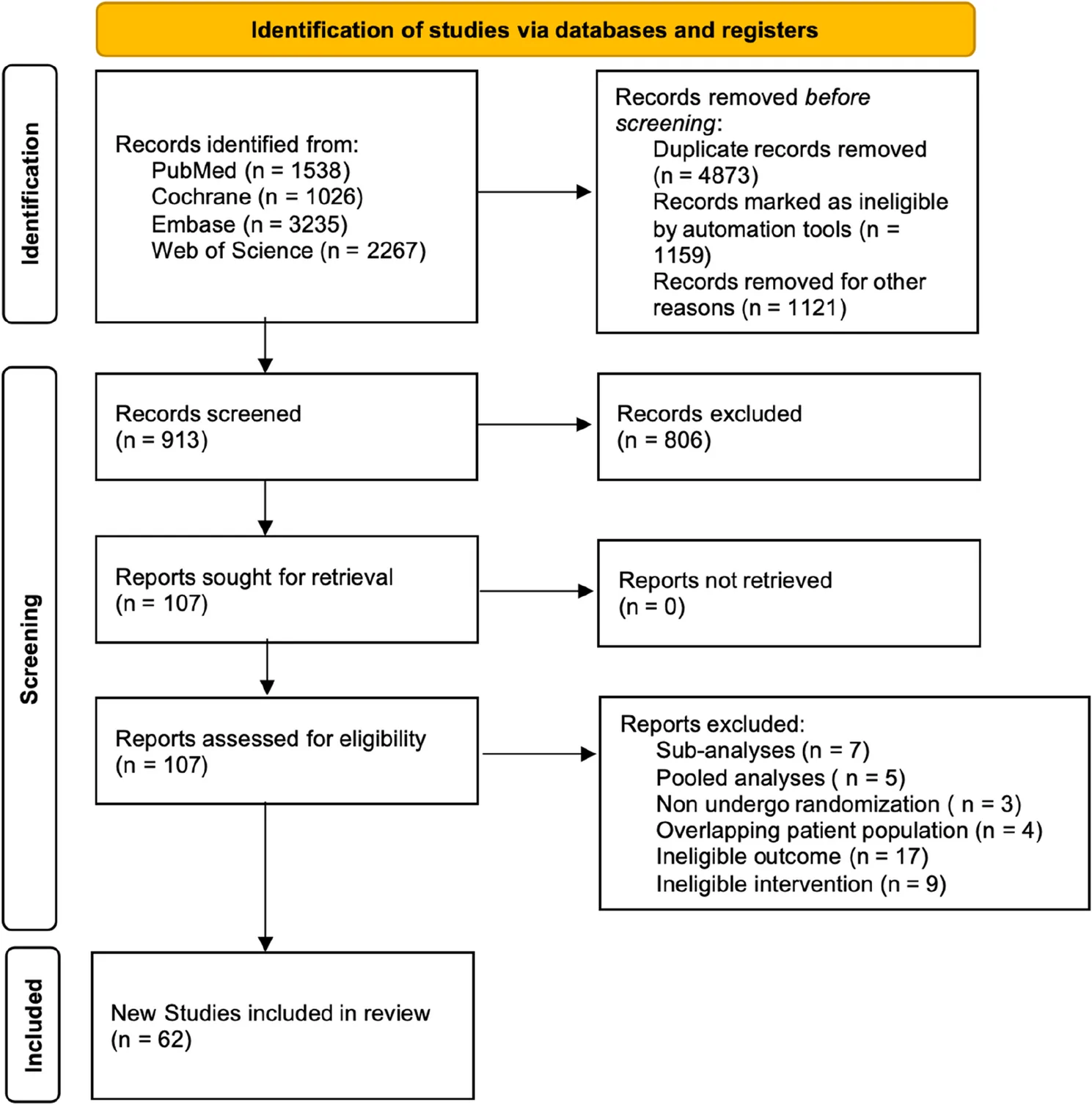
PRISMA flow diagram of the study selection process

The search identified 8066 records. After removal of 4873 duplicates, 1159 records were excluded by automation tools, and 1121 records were excluded for other reasons, 913 records underwent title and abstract screening. Of these, 107 full−text reports were assessed for eligibility. Forty−five reports were excluded because they did not report eligible outcomes, contained overlapping or redundant data, or were not randomized. In total, 62 randomized controlled trials involving 73,240 participants were included in the network meta−analysis (Fig. 1).

The included trials were published between 2012 and 2025 and evaluated six PCSK9 inhibitors: alirocumab (*n* = 21), evolocumab (*n* = 21), inclisiran (*n* = 8), tafolecimab (*n* = 3), recaticimab (*n* = 3), and ongericimab (*n* = 6). Trial populations included adults with hypercholesterolemia, hyperlipidaemia, or mixed dyslipidemia, with or without established ASCVD. Thirty−three trials were multinational, and the remaining studies were conducted in China (*n* = 14), Japan (*n* = 9), the USA (*n* = 5), and Switzerland (*n* = 1). Follow − up ranged from 8 weeks to a median of 2.8 years. Mean or median age ranged from 42.3 to 66.4 years. Seven trials assessed PCSK9 inhibition as monotherapy, usually in statin−intolerant populations or in participants not receiving background lipid−lowering treatment; the other trials mostly used stable statin therapy, with or without ezetimibe, as background treatment. Detailed baseline characteristics for all 62 trials are provided in the Appendix 2 (Table S2.1).

### Risk of bias and certainty of evidence

Risk of bias assessments are shown in Appendix 4. Overall, 58 trials (93.5%) were judged to be at low risk of bias, and 4 trials (6.5%) raised some concerns, mainly because of possible deviations from intended interventions. Between−study heterogeneity was low for safety outcomes (AEs: τ^2^ = 0.01; SAEs: τ^2^ = 0.05), moderate for HDL − C (τ^2^ = 0.15) and high for Lp(a), LDL − C, non − HDL−C, TG, TC, and ApoB. CINeMA assessments indicated low confidence for most pairwise comparisons and very low confidence for several comparisons. Confidence in the estimates was limited primarily by indirectness, with additional concerns regarding imprecision and heterogeneity (Appendix 9). Transitivity was assessed using prespecified clinical effect modifiers, including age, baseline Lp(a), and baseline LDL − C. The distributions of these effect modifiers across treatment nodes are presented in Appendix 9 (Table S9.1). Mean age ranged from 50.11 to 63.51 years, and mean baseline LDL − C ranged from 2.92 to 3.29 mmol/L, indicating broadly similar distributions across treatment nodes. Baseline Lp(a) showed greater variability and was reported in both nmol/L and mg/dL. Among treatment−node summaries based on studies reporting nmol/L, mean baseline Lp(a) ranged from 57.93 to 113.18 nmol/L; among those based on studies reporting mg/dL, mean values ranged from 24.70 to 27.93 mg/dL. Because Lp(a) values expressed in mg/dL and nmol/L cannot be reliably interconverted using a single fixed conversion factor, they were summarized separately according to the original reporting unit. The observed variation in baseline Lp(a) and its reporting methods may have affected comparability across treatment nodes and was considered when interpreting the network estimates. Visual inspection of funnel plots (Appendix 10) showed general symmetry across the outcomes and did not suggest marked small−study effects.

### Primary outcome: Lp(a)

**Fig. 2:**
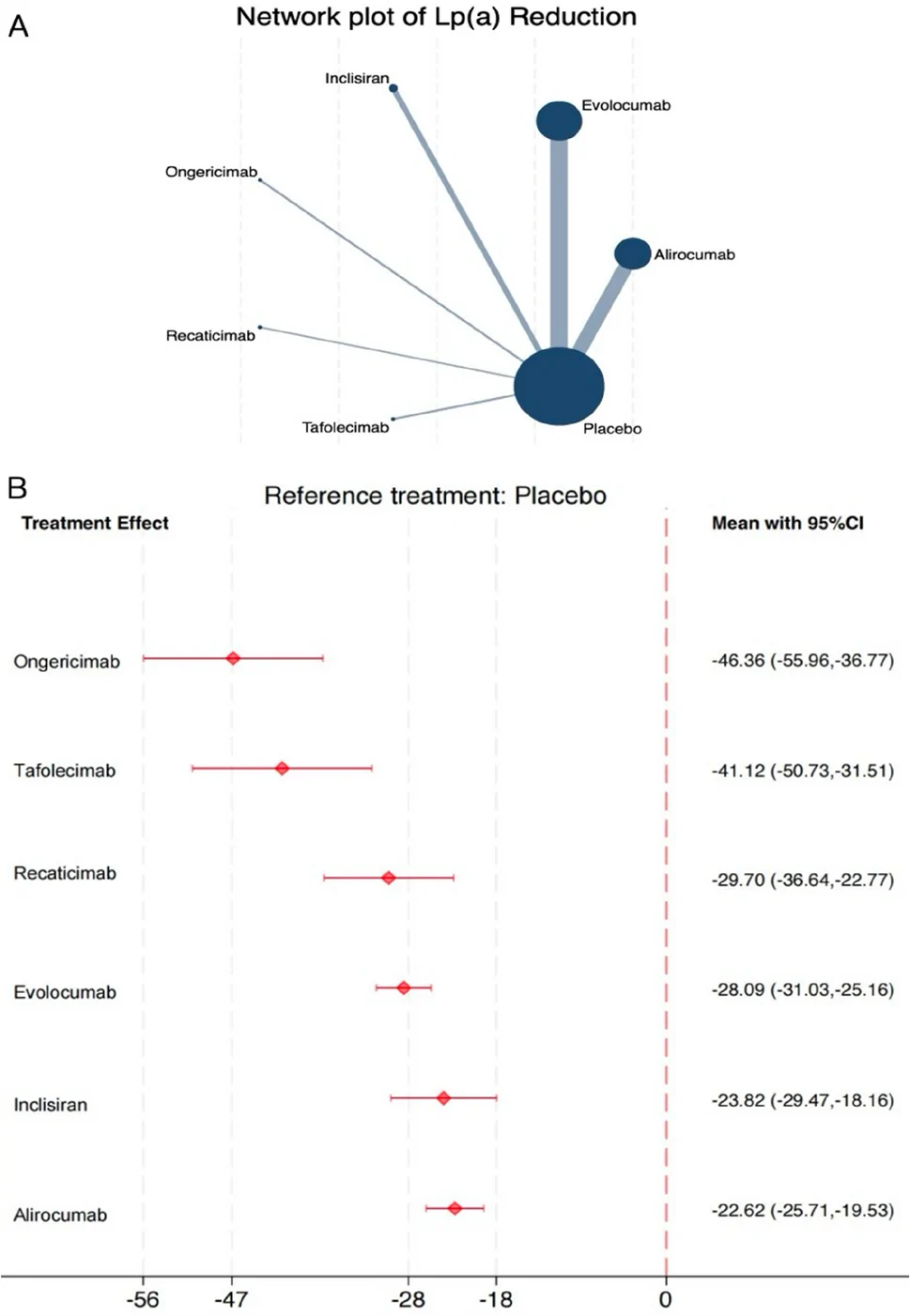
Network plot and forest plot for the reduction in Lp(a). **A** Network plot of treatment comparisons; **B** Forest plot of network meta−analysis relative estimates compared with placebo

The Lp(a) network included seven treatment nodes, with placebo and evolocumab forming the most connected parts of the network (Fig. 2A). All evaluated PCSK9 inhibitors significantly reduced Lp(a) compared with placebo (Fig. 2B). Ongericimab showed the largest point estimate (MD = − 46.36%, 95% CI [− 55.96 to − 36.77], SUCRA = 96.2%, low certainty), followed by tafolecimab (MD = − 41.12%, 95% CI [− 50.73 to − 31.51], SUCRA = 86.5%, low certainty) and recaticimab (MD = − 29.70%, 95% CI [− 36.64 to − 22.77], SUCRA = 59.1%, low certainty). Alirocumab showed the smallest estimated reduction among the active treatments (MD = − 22.62%, 95% CI [− 25.71 to − 19.53], SUCRA = 23.4%, low certainty).

The league table summarized the corresponding between−treatment estimates (Appendix 8, Table S8.1). Ongericimab was associated with greater estimated Lp(a) reductions than alirocumab, evolocumab, inclisiran, and recaticimab, whereas the estimated difference between ongericimab and tafolecimab was not statistically significant. Tafolecimab was also associated with greater estimated reductions than alirocumab, evolocumab, and inclisiran, but the estimated difference from recaticimab was not statistically significant. Evolocumab was associated with a greater estimated Lp(a) reduction than alirocumab (MD = − 5.48%, 95% CI [− 9.74 to − 1.21]). No statistically significant differences were observed among alirocumab, inclisiran, and recaticimab, or between evolocumab and either inclisiran or recaticimab. Because these between−treatment comparisons were based predominantly on indirect evidence, the relative estimates should be interpreted cautiously.

### Secondary lipid outcomes

**Fig. 3:**
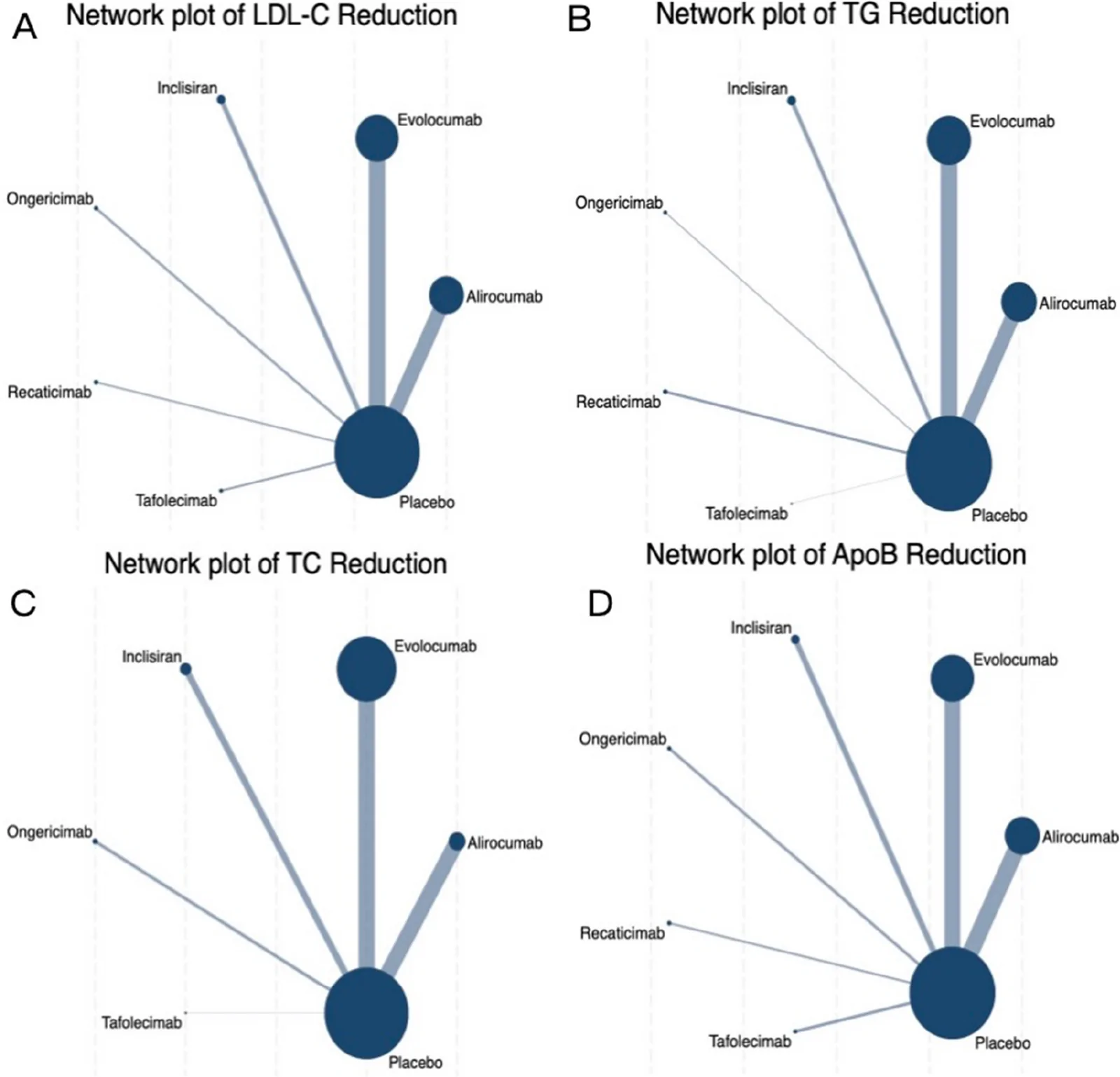
Network plots for secondary lipid parameters. **A** LDL − C; **B** TG; **C** TC; **D** ApoB

**Fig. 4:**
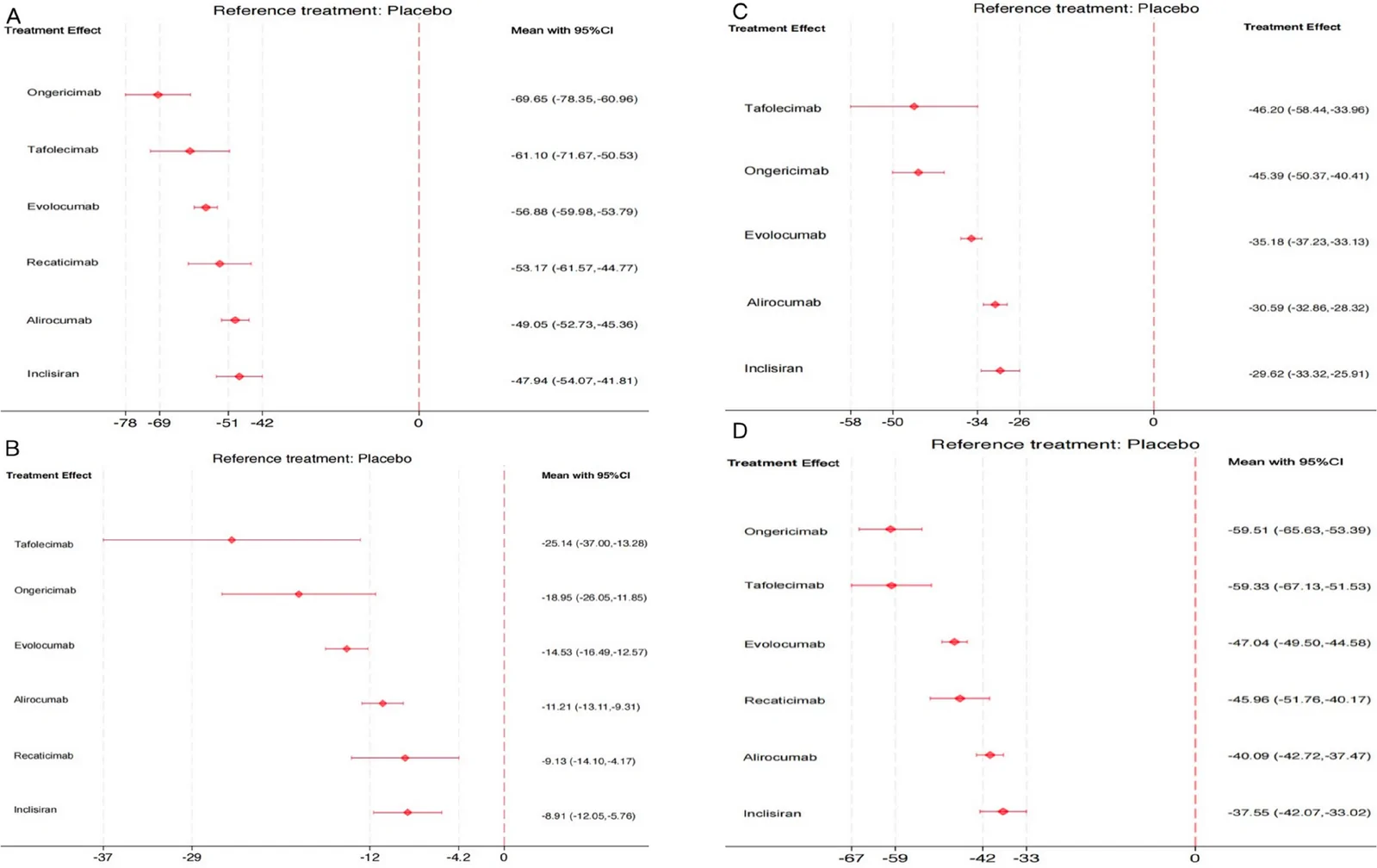
Forest plots of network meta−analysis relative estimates for secondary lipid outcomes. **A **LDL − C; **B** TG; **C** TC; **D** ApoB. All estimates are presented relative to placebo

Network analyses of LDL − C, non − HDL−C, TG, TC, HDL − C, and ApoB were broadly similar in structure to the primary Lp(a) network, although recaticimab was not represented in the TC and HDL − C networks (Fig. 3; Appendix 6). Most evaluated PCSK9 inhibitors were associated with significant improvements in atherogenic lipid profiles relative to placebo (Fig. 4). For LDL − C, ongericimab had the largest point estimate for reduction (MD = − 69.65%, 95% CI [− 78.35 to − 60.96], SUCRA = 98.1%), followed by tafolecimab (MD = − 61.10%, 95% CI [− 71.67 to − 50.53], SUCRA = 78.7%).

Ongericimab and tafolecimab generally showed larger point estimates across the secondary lipid outcomes, although their relative performance varied by outcome. For ApoB, ongericimab (MD = − 59.51%, 95% CI [− 65.63 to − 53.39], SUCRA = 91.8%) and tafolecimab (MD = − 59.33%, 95% CI [− 67.13 to − 51.53], SUCRA = 91.4%) showed the largest estimated reductions. For TG, tafolecimab (MD = − 25.14%, 95% CI [− 37.00 to − 13.28], SUCRA = 95.6%) and ongericimab (MD = − 18.95%, 95% CI [− 26.05 to − 11.85], SUCRA = 84.1%) showed the largest point estimates for reduction. For TC, tafolecimab (MD = − 46.20%, 95% CI [− 58.44 to − 33.96], SUCRA = 90.2%) and ongericimab (MD = − 45.39%, 95% CI [− 50.37 to − 40.41], SUCRA = 88.9%) showed the largest estimated reductions. All evaluated interventions showed numerical increases in HDL − C; however, the effect of tafolecimab was not statistically significant compared with placebo (MD = 4.04%, 95% CI [− 0.36 to 8.44]). Detailed SUCRA rankings and league−table comparisons are provided in Appendices 7 and 8.

The league tables provided the corresponding between−treatment estimates. Ongericimab was associated with greater estimated reductions than alirocumab, evolocumab, and inclisiran in LDL − C, non − HDL−C, TC, and ApoB, and with greater estimated reductions in TG than alirocumab and inclisiran. Tafolecimab was associated with greater estimated reductions than alirocumab and inclisiran across LDL − C, non − HDL−C, TG, TC, and ApoB, and with greater estimated reductions than evolocumab in non − HDL−C and ApoB. No statistically significant differences were observed between ongericimab and tafolecimab for these lipid−lowering outcomes. Evolocumab was associated with greater estimated reductions than alirocumab in LDL − C (MD = − 7.84%, 95% CI [− 12.65 to − 3.02]), non − HDL−C (MD = − 8.75%, 95% CI [− 12.42 to − 5.07]), TG (MD = − 3.32%, 95% CI [− 6.05 to − 0.59]), TC (MD = − 4.59%, 95% CI [− 7.65 to − 1.53]), and ApoB (MD = − 6.95%, 95% CI [− 10.54 to − 3.35]). For HDL − C, ongericimab was associated with a greater estimated increase than alirocumab, evolocumab, inclisiran, and tafolecimab. Detailed league−table comparisons are provided in Appendix 8.

### Safety outcomes

**Fig. 5:**
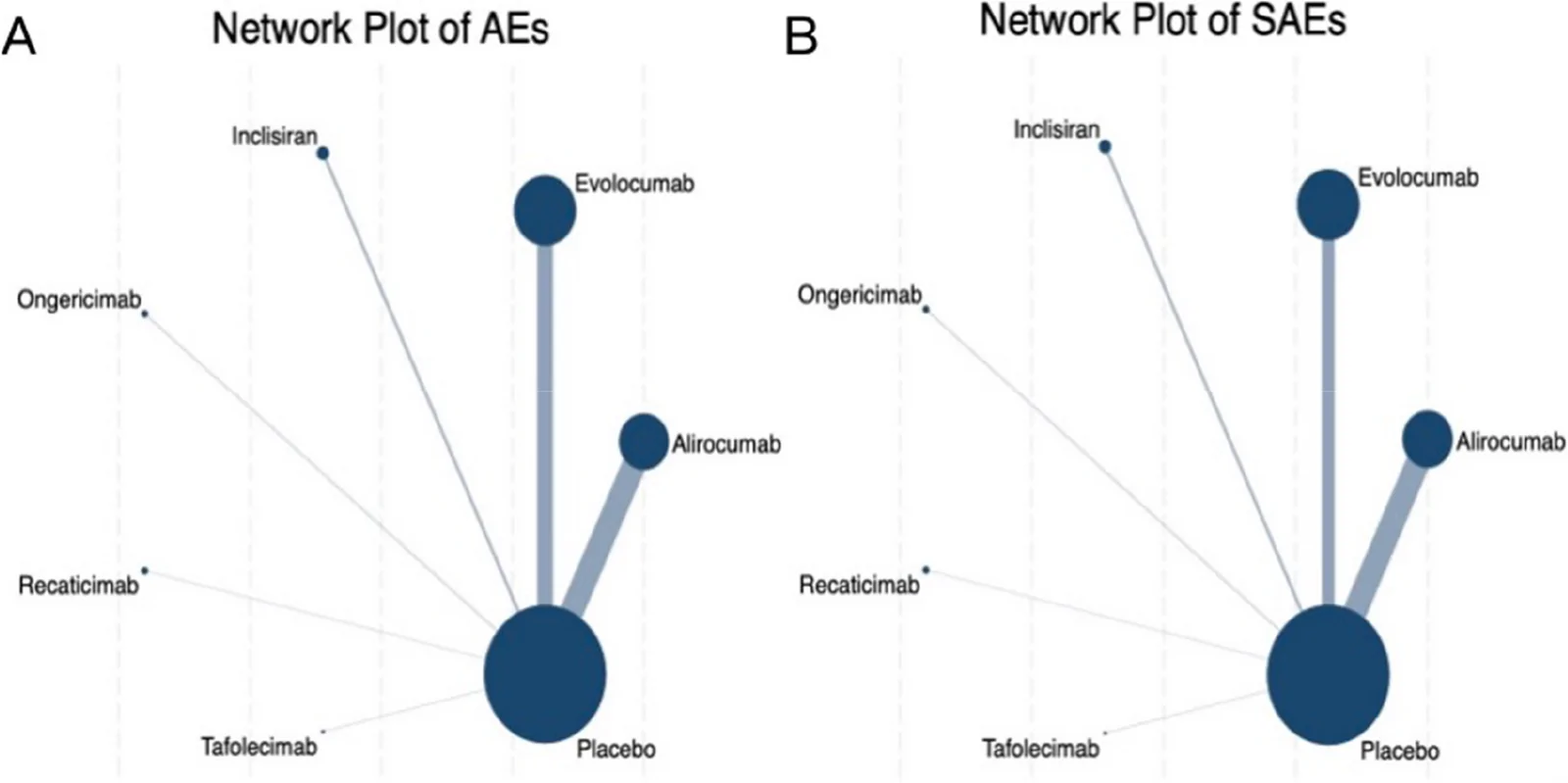
Network plots for safety outcomes. **A **AEs; **B** SAEs

**Fig. 6:**
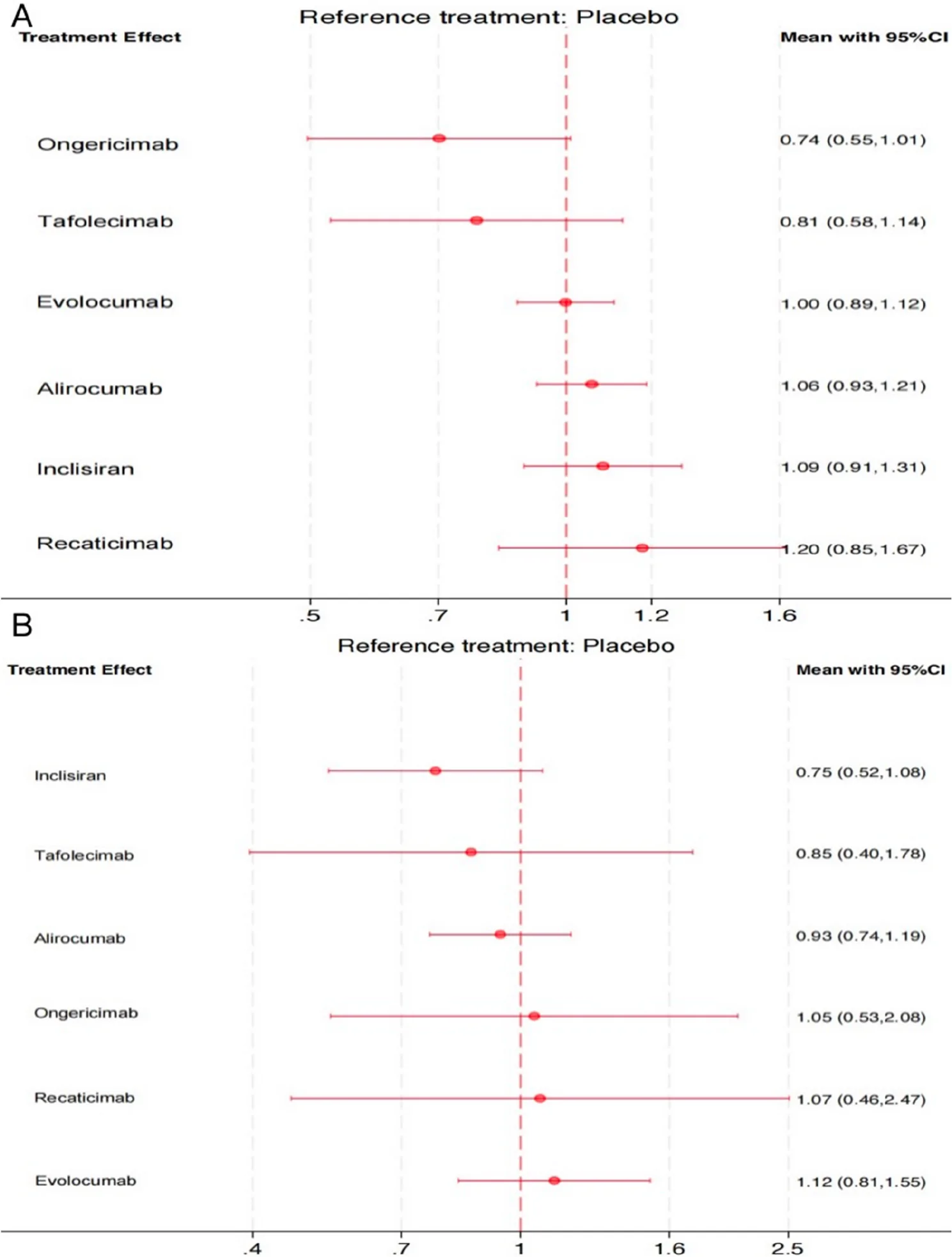
Forest plots of network meta−analysis relative estimates for safety outcomes. **A** AEs; **B** SAEs. All estimates are presented relative to placebo

The safety networks for AEs and SAEs had a topology similar to that of the efficacy networks, with placebo and evolocumab as the most connected nodes (Fig. 5). No statistically significant differences in the risk of AEs were observed between any evaluated PCSK9 inhibitor and placebo; ORs ranged from 0.74 to 1.20, and all 95% CIs included 1 (Fig. 6A). Ongericimab had the lowest point estimate for AEs (OR = 0.74, 95% CI [0.55 to 1.01]), although the difference from placebo was not statistically significant. Pairwise comparisons in the league table suggested higher estimated odds of AEs with alirocumab (OR = 1.43, 95% CI [1.02 to 2.00]) and inclisiran (OR = 1.47, 95% CI [1.02 to 2.10]) than with ongericimab. Ongericimab was also associated with lower estimated odds of AEs than recaticimab (OR = 0.62, 95% CI [0.39 to 0.98]), whereas no other between−treatment differences were statistically significant (Table S8.8).

For SAEs, no statistically significant differences were observed between any active treatment and placebo (Fig. 6B). ORs ranged from 0.75 for inclisiran to 1.12 for evolocumab, with all 95% CIs including 1. Pairwise comparisons in the league table also showed no statistically significant differences between the active PCSK9 inhibitors (Table S8.9).

Specific AEs were inconsistently reported across the included trials (Appendix 16). Injection−site reactions were among the most frequently reported events and occurred more often in the treatment groups in several trials, although their frequencies varied substantially across studies and agents. Muscle−related events, hepatic effects, neurocognitive events, and diabetes or glucose−control events showed no consistent directional pattern between intervention and comparator groups. Nasopharyngitis, upper respiratory tract infection, urinary tract infection, and headache were also variably reported. Because event definitions, ascertainment methods, and reporting completeness differed across trials, these outcomes were summarized descriptively and were not quantitatively pooled.

### Subgroup analyses

Follow − up duration was categorised as early response (≤ 12 weeks), mid−term maintenance (13–26 weeks), and long−term follow − up (> 26 weeks) (Appendix 12). During the early phase, recaticimab showed the largest point estimate for Lp(a) reduction (MD = − 36.90%, 95% CI [− 57.70 to − 16.10]), followed by tafolecimab (MD = − 36.40%, 95% CI [− 50.67 to − 22.13]). During weeks 13–26, ongericimab showed the largest point estimate for reduction (MD = − 50.65%, 95% CI [− 61.42 to − 39.89]). Among studies with follow − up longer than 26 weeks, tafolecimab showed the largest point estimate for reduction (MD = − 46.07%, 95% CI [− 57.36 to − 34.78]). Thus, the treatment with the largest point estimate differed across follow − up intervals. These subgroup findings were exploratory and should not be interpreted as evidence of differential treatment effects over time.

Analyses stratified by background statin therapy showed differences in the estimated Lp(a) reductions for some agents. For example, ongericimab showed a larger point estimate for reduction among participants receiving background statins (MD = − 50.87%, 95% CI [− 62.29 to − 39.46]) than among those not receiving statins (MD = − 33.50%, 95% CI [− 45.22 to − 21.78]). However, these subgroup estimates may also reflect differences in trial populations, baseline characteristics, and study design and should therefore be interpreted cautiously.

Geographical analyses compared East Asian cohorts with global populations. East Asian studies included trials conducted in China, Japan, South Korea, and Singapore. Ongericimab (MD = − 45.37%, 95% CI [− 53.07 to − 37.68]) and tafolecimab (MD = − 41.29%, 95% CI [− 48.98 to − 33.60]) showed large estimated reductions in Lp(a) in East Asian populations, but these estimates were mainly derived from trials conducted in East Asia. Evolocumab showed a larger point estimate for reduction in East Asian cohorts (MD = − 38.48%, 95% CI [− 44.34 to − 32.61]) than in global cohorts (MD = − 26.14%, 95% CI [− 28.77 to − 23.51]). Nevertheless, the regional pattern was not consistent across all agents, and these exploratory findings should not be interpreted as definitive evidence of regional differences in treatment response.

### Sensitivity analyses

Sensitivity analyses were broadly consistent with the primary findings. When the analysis was restricted to label−approved dose regimens, all evaluated regimens significantly reduced Lp(a) compared with placebo. Ongericimab 150 mg Q2W showed the largest point estimate for reduction (MD = − 46.03%, 95% CI [− 58.21 to − 33.85]), followed by ongericimab 300 mg Q4W (MD = − 44.80%, 95% CI [− 62.21 to − 27.39]) and tafolecimab 150 mg Q2W (MD = − 43.30%, 95% CI [− 69.79 to − 16.81]). The remaining label−approved regimens also produced statistically significant reductions, indicating that restriction to label−approved regimens did not materially alter the direction of the primary findings.

In the reporting−unit–based sensitivity analysis, among studies reporting Lp(a) in mg/dL, tafolecimab showed the largest point estimate for reduction (MD = − 41.05%, 95% CI [− 51.76 to − 30.33]), followed by evolocumab (MD = − 34.93%, 95% CI [− 47.12 to − 22.74]) and alirocumab (MD = − 22.95%, 95% CI [− 26.53 to − 19.36]). Among studies reporting Lp(a) in nmol/L, ongericimab showed the largest point estimate for reduction (MD = − 45.93%, 95% CI [− 54.59 to − 37.26]), followed by recaticimab (MD = − 29.66%, 95% CI [− 35.75 to − 23.58]), evolocumab (MD = − 27.49%, 95% CI [− 30.18 to − 24.79]), and inclisiran (MD = − 23.63%, 95% CI [− 28.66 to − 18.61]). Alirocumab showed a numerical reduction in the nmol/L stratum, but the difference from placebo was not statistically significant (MD = − 19.25%, 95% CI [− 40.48 to 1.99]). Overall, the direction of the estimates was broadly consistent with the primary analysis, although the treatments represented differed between the two reporting−unit strata. The relative magnitudes observed in the two strata should therefore not be compared directly.

## Discussion

This network meta−analysis compared the lipid−lowering efficacy and safety of PCSK9 inhibitors in adults with hypercholesterolemia or dyslipidemia, incorporating 62 randomized trials and 73,240 participants. The analysis included established monoclonal antibodies, the small interfering RNA agent inclisiran, and newer agents including tafolecimab, recaticimab, and ongericimab. Compared with placebo, all evaluated PCSK9 inhibitors reduced Lp(a), the primary endpoint of this study. Ongericimab showed the largest point estimate for Lp(a) reduction, followed by tafolecimab. Similar patterns were observed for several atherogenic lipid measures, particularly LDL − C and ApoB, for which ongericimab and tafolecimab generally showed larger point estimates than the other evaluated agents. However, these comparative estimates were based predominantly on indirect evidence and were rated as having low or very low certainty. No statistically significant differences in overall AEs or SAEs were detected between the evaluated PCSK9 inhibitors and placebo, although this should not be interpreted as establishing equivalent safety across agents.

Previous meta−analyses have mainly focused on established PCSK9 inhibitors, particularly evolocumab and alirocumab, and have consistently reported Lp(a) reductions of approximately 20% to 30% [35, 36]. Our findings are consistent with this evidence for conventional monoclonal antibodies, while extending the evidence base by incorporating newer agents, including tafolecimab, recaticimab, and ongericimab, into a single comparative network. The larger point estimates for Lp(a) reduction observed for ongericimab and tafolecimab are broadly consistent with a recent network meta−analysis in Chinese populations by Wang et al. [37], in which ongericimab 150 mg every two weeks showed relatively large estimated effects across several lipid endpoints and was associated with a 50.93% reduction in Lp(a). The present study differs from previous pairwise meta−analyses in two important respects. First, it compared multiple PCSK9 inhibitors simultaneously, allowing indirect estimation of relative efficacy among agents that have not been tested head to head. Second, it evaluated Lp(a) alongside LDL − C, TC, TG, ApoB and safety outcomes, thereby placing Lp(a) lowering within the broader lipid−modifying profile of each agent. This distinction is clinically relevant because PCSK9 inhibitors reduce several ApoB−containing lipoproteins at the same time, and the observed cardiovascular benefit of this drug class cannot be attributed to Lp(a) lowering alone.

Lp(a) is a genetically determined and independent risk factor for ASCVD, and elevated concentrations contribute to residual cardiovascular risk even when LDL − C is well controlled [38, 39]. In this context, the consistent Lp(a) reductions observed in our analysis represent a potentially relevant lipid−biomarker effect. However, Lp(a) was a surrogate endpoint, and the present findings do not demonstrate that Lp(a) lowering itself reduces cardiovascular events. PCSK9 inhibitors produce substantial reductions in LDL − C and ApoB, which are established causal drivers of ASCVD risk [40, 41]. Large outcome trials of PCSK9 inhibitors have shown cardiovascular benefit, but much of that benefit is likely mediated through LDL − C and ApoB lowering rather than through Lp(a) reduction alone. Considerably larger absolute reductions in Lp(a) may be required to achieve meaningful cardiovascular risk reduction than the modest Lp(a) lowering typically observed with currently available PCSK9 inhibitors [42]. Therefore, our findings should be interpreted as evidence that PCSK9 inhibitors improve Lp(a) as part of a broader lipid−lowering profile, not as proof that Lp(a) lowering by these agents independently improves cardiovascular outcomes. Dedicated cardiovascular outcome trials of Lp(a)−targeted therapies are required to establish whether pharmacological Lp(a) reduction translates into clinical benefit.

The use of percentage change in Lp(a) also deserves emphasis. Because baseline Lp(a) varies substantially across populations and is strongly genetically determined, absolute reductions are influenced by baseline concentrations. Percentage change avoids the need for direct conversion between mg/dL and nmol/L and may facilitate cross−trial synthesis. However, it does not eliminate heterogeneity arising from differences in assay platforms, calibration methods, apo(a) isoform sensitivity, or baseline Lp(a) concentrations. Similar relative reductions may therefore correspond to substantially different absolute reductions and post-treatment concentrations. Patients with very high baseline Lp(a) may remain above risk−enhancing thresholds even after a large relative reduction. Although the reporting-unit–based sensitivity analyses yielded broadly similar directions of effect, residual measurement-related heterogeneity cannot be excluded.

Observed reductions in Lp(a), LDL − C, and ApoB are biologically plausible but do not confirm a mechanistic pathway. PCSK9 inhibition increases hepatic LDL receptor availability by reducing PCSK9 − mediated receptor degradation, thereby enhancing LDL − C clearance [43]. Lp(a) contains an ApoB − 100 component and may be partly affected by pathways related to LDL receptor activity, hepatic particle handling, or Apo(a)−containing particle clearance [44]. However, the exact mechanisms underlying PCSK9 inhibitor−mediated Lp(a) reduction remain incompletely defined.

The relatively large point estimates observed for ongericimab and tafolecimab may partly reflect differences in lipid-lowering activity, but several alternative explanations should be considered. Trials of newer agents differed from those of established agents in geography, baseline lipid profiles, background therapy, dose regimens, follow − up duration, and Lp(a) measurement methods. Moreover, the evidence for these newer agents was derived from fewer trials and was geographically concentrated, particularly in East Asian populations. Although subgroup evaluations were employed to address these variations, the intrinsic differences in trial−level characteristics necessitate a cautious interpretation of the hierarchy among newer agents. Accordingly, the current rankings should be viewed as exploratory rather than as definitive evidence of comparative superiority.

Subgroup analyses suggested that the magnitude of the estimated Lp(a) reduction varied according to follow − up duration, background statin therapy, and geographical region. These analyses were exploratory, and differences between subgroup−specific point estimates may reflect variation in study populations, baseline characteristics, follow − up schedules, and trial design rather than true effect modification. In the East Asian subgroup, established agents such as evolocumab also showed a greater estimated Lp(a) reduction versus placebo than in the global cohort (MD = − 38.48% vs. −26.14%). Ongericimab and tafolecimab also showed relatively large reductions in East Asian studies (MD = − 45.37% and − 41.29%, respectively). However, these estimates were derived mainly from trials conducted within the region and should not be interpreted as evidence that East Asian populations respond more favorably to these agents. In the sensitivity analysis restricted to label−approved dose regimens, the direction of the findings was broadly consistent with the primary analysis, suggesting that the main results were not driven solely by nonstandard dose regimens. Nevertheless, pooling different doses and administration schedules within drug−level nodes in the primary analysis may still have contributed to heterogeneity and uncertainty in treatment rankings.

The comparison between monoclonal antibodies and inclisiran also requires caution. Monoclonal antibodies directly bind circulating PCSK9, whereas inclisiran reduces hepatic PCSK9 synthesis through RNA interference [45, 46]. These approaches differ in pharmacodynamic trajectory, dosing interval, and timing of maximal effect. A single trial endpoint may not fully capture these differences. Therefore, apparent differences between inclisiran and monoclonal antibodies may reflect assessment timing and trial design as much as intrinsic differences in lipid−lowering capacity.

### Strengths and limitations of this study

This study provides an updated comparative synthesis of both widely used and newer PCSK9 inhibitors, with Lp(a) as the prespecified primary outcome and multiple lipid and safety endpoints assessed within the same network. The use of a prespecified protocol, duplicate data extraction, RoB 2 assessment, transitivity assessment, sensitivity analyses, and CINeMA certainty appraisal strengthens the credibility and transparency of the findings. Several limitations should nevertheless be acknowledged. First, direct head − to−head trials were scarce, especially for comparisons between newer agents and established monoclonal antibodies; therefore, most treatment comparisons and treatment rankings relied predominantly on indirect evidence, with low or very low certainty. Second, the evidence for newer agents was based on relatively few, geographically concentrated trials, which may limit generalizability beyond the populations studied. Third, different dose regimens and administration schedules were combined within drug−level nodes in the primary analysis. Although the sensitivity analysis restricted to label−approved regimens produced broadly similar findings, residual dose−related heterogeneity may have affected the estimated hierarchy. Fourth, Lp(a) assays, reporting units, baseline concentrations, and measurement methods varied across trials. The reporting−unit–based sensitivity analysis could not fully account for differences in assay platforms, calibration, or apo(a) isoform sensitivity. Fifth, the lack of granular baseline data in many original reports restricted our ability to perform more detailed subgroup analyses on specific clinical factors, such as different genetic backgrounds or baseline PCSK9 levels, which could further explain the observed heterogeneity. Sixth, heterogeneity was substantial for several lipid outcomes, including Lp(a), LDL − C, TC, and ApoB, indicating that between−study differences should be considered when interpreting the hierarchy. Seventh, SUCRA rankings should also be interpreted cautiously because they do not convey the magnitude or precision of treatment differences and may be unstable when treatment nodes are informed by few studies or when confidence intervals overlap substantially. Eighth, specific AEs were inconsistently defined and incompletely reported across trials, precluding reliable quantitative pooling and limiting comparisons of individual safety outcomes. Finally, as several newer PCSK9 inhibitors are in the early stages of clinical application, the current evidence is primarily focused on lipid−lowering efficacy. Consequently, comparative long−term cardiovascular outcomes could not be evaluated, and the present analysis cannot establish whether the observed Lp(a) reductions independently translate into cardiovascular benefit.

## Conclusions

In summary, PCSK9 inhibitors were associated with reductions in Lp(a) and improvements in atherogenic lipid profiles compared with placebo. Comparative differences among individual agents remain uncertain because the evidence was predominantly indirect and of low or very low certainty. No statistically significant differences in overall AEs or SAEs were detected versus placebo. Whether Lp(a) lowering itself improves cardiovascular outcomes remains unknown.

## Supplementary Information

Supplementary Material 1.

Supplementary Material 2.

## Data Availability

The raw datasets supporting the findings of this study were derived from the original peer-reviewed publications identified in our search. The compiled dataset generated and analyzed during this network meta-analysis is included in the Supplementary Materials. Comprehensive details regarding the study methodology are documented in the PROSPERO registry (CRD420251208404) and further elaborated in the Supplementary Materials accompanying this article.

## Abbreviations

AE: Adverse event
Apo(a): Apolipoprotein(a)
ApoB: Apolipoprotein B
ASCVD: Atherosclerotic cardiovascular disease
BC: Box − Cox
CENTRAL: Cochrane Central Register of Controlled Trials
CI: Confidence interval
CINeMA: Confidence in Network Meta−Analysis
HDL − C: High−density lipoprotein cholesterol
IQR: Interquartile range
LDL: Low−density lipoprotein
LDL − C: Low−density lipoprotein cholesterol
LDLR: Low−density lipoprotein receptors
Lp(a): Lipoprotein(a)
MD: Mean Difference
MeSH: Medical Subject Headings
NMA: Network meta−analysis
Non − HDL−C: Non−high−density lipoprotein cholesterol
OR: Odds ratio
PCSK9: Proprotein convertase subtilisin/kexin type 9
PD: Pharmacodynamic
PK: Pharmacokinetic
PRISMA: Preferred Reporting Items for Systematic Reviews and Meta−Analyses
QE: Quantile Estimation
RCT: Randomized controlled trial
SAE: Serious adverse event
SD: Standard deviation
SE: Standard error
siRNA: Small interfering RNA
SUCRA: Surface under the cumulative ranking
TC: Total cholesterol
TG: Triglycerides
