# Supplementary material for: Comparative efficacy and safety of PCSK9 inhibitors for lipoprotein(a) reduction: a systematic review and network meta−analysis
Source: BMC Cardiovasc Disord. 2026 Aug 18;26:1073. doi: 10.1186/s12872-026-06480-7 (PMC13756961; doi:10.1186/s12872-026-06480-7)
Supplement: Supplementary file 1 [file 12872_2026_6480_MOESM1_ESM.docx]

**Supplementary Appendix**

**Comparative Efficacy and Safety of PCSK9 Inhibitors for**

**Lipoprotein(a) Reduction: A Systematic Review and Network Meta-Analysis**

**Contents**

[Appendix 1: Search strategy	3](#_Toc1113391048)

[Table S1.1. Search strategy of PubMed	3](#_Toc1738327425)

[Table S1.2. Search strategy of Web of Science: Science Citation Index Expanded	4](#_Toc1701498187)

[Table S1.3. Search strategy of Embase	4](#_Toc1187785457)

[Table S1.4. Search strategy of Cochrane Central Register of Controlled Trials	5](#_Toc102193287)

[(CENTRAL)	5](#_Toc1723140656)

[Appendix 2: Characteristics of included studies	6](#_Toc2008025597)

[Table S2.1: Baseline of characteristics of included studies	6](#_Toc1180696174)

[References	13](#_Toc1211698138)

[Table S2.2: PCSK9 inhibitors list	24](#_Toc423180865)

[Appendix 3: List of data extracted from the included randomized clinical trials	26](#_Toc2082442838)

[Appendix 4: Risk of bias of randomized clinical trials	27](#_Toc2075783107)

[Figure S4.1: Overall risk of bias presented as percentage of each risk of bias item across all included studies.	27](#_Toc1814833834)

[Table S4.2: Study level risk of bias assessment using Cochrane risk of bias tool 2.0 for assessing risk of bias of randomized clinical trials.	28](#_Toc1202009697)

[Appendix 5: Evaluation of heterogeneity	32](#_Toc798310150)

[Table S5.1: Network heterogeneity	32](#_Toc1868348241)

[Appendix 6: Network plots and forest plots of secondary outcomes	33](#_Toc823000053)

[Figure S6.1: Network plot of the effect on non-HDL-C, and forest plot of network effect sizes for compared with placebo.	33](#_Toc219720444)

[Appendix 7: SUCRA and cumulative probability plots	35](#_Toc1317113115)

[Figure S7.1: Cumulative ranking curve plots of PCSK9 inhibitors for Lp(a) in range network.	35](#_Toc458690529)

[Figure S7.2: Cumulative ranking curve plots of PCSK9 inhibitors for LDL-C in range network.	36](#_Toc1892911820)

[Figure S7.3: Cumulative ranking curve plots of PCSK9 inhibitors for non-HDL-C in range network.	37](#_Toc1346212082)

[Figure S7.4: Cumulative ranking curve plots of PCSK9 inhibitors for TG in range network.	38](#_Toc2046241029)

[Figure S7.5: Cumulative ranking curve plots of PCSK9 inhibitors for TC in range network.	39](#_Toc1369851345)

[Figure S7.6: Cumulative ranking curve plots of PCSK9 inhibitors for HDL-C in range network.	40](#_Toc2066859575)

[Figure S7.7: Cumulative ranking curve plots of PCSK9 inhibitors for ApoB in range network.	41](#_Toc13403153)

[Figure S7.8: Cumulative ranking curve plots of PCSK9 inhibitors for AE in range network.	42](#_Toc1928493183)

[Figure S7.9: Cumulative ranking curve plots of PCSK9 inhibitors for SAE in range network.	43](#_Toc214242510)

[Appendix 8: League table of Summary Estimates for PCSK9 inhibitors	44](#_Toc1591273198)

[Table S8.1: Lp(a)	44](#_Toc1914782695)

[Table S8.2: LDL-C	45](#_Toc1710304570)

[Table S8.3: non-HDL-C	46](#_Toc1020292895)

[Table S8.4: TG	47](#_Toc405764970)

[Table S8.5: TC	48](#_Toc1431271565)

[Table S8.6: HDL-C	49](#_Toc1416862908)

[Table S8.7: ApoB	50](#_Toc1916216820)

[Table S8.8: AEs	51](#_Toc43839681)

[Table S8.9: SAEs	52](#_Toc226627646)

[Appendix 9: CINeMA Assessment	53](#_Toc1442340191)

[Figure S9.1: Risk of bias contribution by intervention group in Lp(a).	53](#_Toc616182801)

[Figure S9.2: Overall risk of bias by treatment comparison in Lp(a).	54](#_Toc1018190573)

[Figure S9.3: Risk of bias contribution by intervention group in LDL-C.	55](#_Toc1579261115)

[Figure S9.4: Overall risk of bias by treatment comparison in LDL-C.	56](#_Toc1891166532)

[Table S9.1: Transitivity assessment.	57](#_Toc2077927724)

[Table S9.2: CINeMA Results of Lp(a).	58](#_Toc1352189754)

[Table S9.3: CINeMA Results of LDL-C.	60](#_Toc1581242924)

[Appendix 10: Funnel plots	61](#_Toc839692043)

[Figure S10.1: Funnel plot of Lpa.	61](#_Toc1589122264)

[Figure S10.2: Funnel plot of LDL-C.	61](#_Toc123773309)

[Figure S10.3: Funnel plot of non-HDL-C.	62](#_Toc1493834067)

[Figure S10.4: Funnel plot of TG.	63](#_Toc637846992)

[Figure S10.5: Funnel plot of TC.	63](#_Toc56028720)

[Figure S10.6: Funnel plot of HDL-C.	64](#_Toc1076859654)

[Figure S10.7: Funnel plot of ApoB.	65](#_Toc1935511509)

[Figure S10.8: Funnel plot of AE.	65](#_Toc59647007)

[Figure S10.9: Funnel plot of SAE.	66](#_Toc1759867147)

[Appendix 11: Comprehensive comparisons of different PCSK9 inhibitors at various doses and dosing regimens	67](#_Toc794869498)

[Figure S11.1: Lp(a)	67](#_Toc2023368546)

[Appendix 12: Subgroup analysis of PCSK9 inhibitors stratified by Lp(a) efficacy analysis time points	68](#_Toc1351602377)

[Figure S12.1: Early response (≤12 weeks)	68](#_Toc299132273)

[Figure S12.2: Mid-term maintenance (13–26 weeks)	69](#_Toc256894684)

[Figure S12.3: Long-term durability (>26 weeks)	70](#_Toc1186823518)

[Appendix 13: Subgroup analysis of PCSK9 inhibitors stratified by background statin therapy	71](#_Toc1114753690)

[Figure S13.1: With background statins	71](#_Toc1017931402)

[Figure S13.2: Without background statins	72](#_Toc1518341412)

[Appendix 14: Comparison of the effects of PCSK9 inhibitors on Lp(a) levels between East Asian and Global populations	73](#_Toc215934183)

[Figure S14.1: East Asian	73](#_Toc2105933898)

[Figure S14.2: Global	74](#_Toc1753037479)

[Appendix 15: Sensitivity analysis	75](#_Toc1972756360)

[Table S15.1 Sensitivity analysis of treatment effects on Lp(a) according to the reported measurement unit (mg/dL vs. nmol/L)	75](#_Toc1116116487)

[Figure S15.1 Sensitivity analysis of treatment effects on Lp(a) restricted to standard approved doses	76](#_Toc300140464)

[Appendix 16: Specific AEs	77](#_Toc21691645)

# **Appendix 1: Search strategy**

## Table S1.1. Search strategy of PubMed

\#	Searches

1	((Hypercholesterolemia[MeSH Terms]) OR (Hyperlipidemias[MeSH Terms])) OR (Dyslipidemias[MeSH Terms])

2	((((hypercholesterolemia[Title/Abstract]) OR (hypercholesterolaemia[Title/Abstract])) OR (hyperlipidemia[Title/Abstract])) OR (hyperlipidaemia[Title/Abstract])) OR (dyslipidemia[Title/Abstract]) OR (dyslipidemia[Title/Abstract])

3	1 OR 2

4	((("PCSK9 Inhibitors"[MeSH Terms]) OR ("Proprotein Convertase 9"[MeSH Terms])) OR ("PCSK9 inhibitor*"[Title/Abstract])) OR ("proprotein convertase subtilisin/kexin type 9"[Title/Abstract])

5 (((alirocumab[Title/Abstract]) OR (praluent[Title/Abstract])) OR (REGN727[Title/Abstract])) OR (SAR236553[Title/Abstract])

6 ((evolocumab[Title/Abstract]) OR (repatha[Title/Abstract])) OR (AMG145[Title/Abstract])

7 ((((Inclisiran[Title/Abstract]) OR (leqvio[Title/Abstract])) OR (ALN60212[Title/Abstract])) OR (ALN-PCS[Title/Abstract])) OR (ALN-PCSsc[Title/Abstract])

8	(tafolecimab[Title/Abstract]) OR (sintbilo[Title/Abstract])

9	(recaticimab[Title/Abstract]) OR (SHR-1209[Title/Abstract])

10	(ongericimab[Title/Abstract]) OR (JS002[Title/Abstract])

11	4 OR 5 OR 6 OR 7 OR 8 OR 9 OR 10

12 ((((((("randomized controlled trial"[Publication Type]) OR ("controlled clinical trial"[Publication Type])) OR (randomized[Title/Abstract])) OR (randomised[Title/Abstract])) OR (randomization[Title/Abstract])) OR (randomly[Title/Abstract])) OR (rct[Title/Abstract])) OR (trial[Title/Abstract])

13 animals[MeSH Terms] NOT humans[MeSH Terms]

14 12 NOT 13

15 3 AND 11 AND 14

## Table S1.2. Search strategy of Web of Science: Science Citation Index Expanded

\#	Searches

1	TS=(hypercholesterolemia OR hypercholesterolaemia OR hyperlipidemia OR hyperlipidaemia OR dyslipidemia OR dyslipidemia)

2	TS=("PCSK9 Inhibitor*" OR "Proprotein Convertase 9" OR "proprotein convertase subtilisin/kexin type 9")

3	TS=(alirocumab OR praluent OR REGN727 OR SAR236553)

4	TS=(evolocumab OR repatha OR AMG145)

5	TS=(Inclisiran OR leqvio OR ALN60212 OR ALN-PCS OR ALN-PCSsc)

6	TS=(tafolecimab OR sintbilo)

7	TS=(recaticimab OR SHR-1209)

8	TS=(ongericimab OR JS002)

9 #2 OR #3 OR #4 OR #5 OR #6 OR #7 OR #8

10 TS=("randomized controlled trial" OR "controlled clinical trial" OR randomized OR randomised OR randomization OR randomly OR rct OR trial)

11 TS=(animal* NOT human*)

12 #10 NOT #11

13 #1 AND #9 AND #12

## Table S1.3. Search strategy of Embase

\#	Searches

1	'hypercholesterolemia'/exp OR 'hyperlipidemia'/exp OR 'dyslipidemia'/exp OR 'hypercholesterolemia':ti,ab,kw OR 'hypercholesterolaemia':ti,ab,kw OR 'hyperlipidemia':ti,ab,kw OR 'hyperlipidaemia':ti,ab,kw OR 'dyslipidemia':ti,ab,kw OR 'dyslipidemia':ti,ab,kw

2	'pcsk9 inhibitor'/exp OR 'proprotein convertase 9'/exp OR 'pcsk9 inhibitor*':ti,ab,kw OR 'proprotein convertase subtilisin/kexin type 9':ti,ab,kw

3	'alirocumab'/exp OR 'praluent':ti,ab,kw OR 'regn727':ti,ab,kw OR 'sar236553':ti,ab,kw

4	'evolocumab'/exp OR 'repatha':ti,ab,kw OR 'amg145':ti,ab,kw

5	'inclisiran'/exp OR 'leqvio':ti,ab,kw OR 'aln60212':ti,ab,kw OR 'aln-pcs':ti,ab,kw OR 'aln-pcssc':ti,ab,kw

6	'tafolecimab'/exp OR 'sintbilo':ti,ab,kw

7	'recaticimab'/exp OR 'shr-1209':ti,ab,kw

8	'ongericimab'/exp OR 'js002':ti,ab,kw

9	#2 OR #3 OR #4 OR #5 OR #6 OR #7 OR #8

10 'randomized controlled trial'/exp OR 'controlled clinical trial'/exp OR 'randomized':ti,ab,kw OR 'randomised':ti,ab,kw OR 'randomization':ti,ab,kw OR 'randomly':ti,ab,kw OR 'rct':ti,ab,kw OR 'trial':ti,ab,kw

11 'animal'/exp NOT 'human'/exp

12 #10 NOT #11

13 #1 AND #9 AND #12

## Table S1.4. Search strategy of Cochrane Central Register of Controlled Trials

## (CENTRAL)

\#	Searches

1	MeSH descriptor: [Hypercholesterolemia] explode all trees

2	MeSH descriptor: [Hyperlipidemias] explode all trees

3	MeSH descriptor: [Dyslipidemias] explode all trees

4	(hypercholesterolemia OR hypercholesterolaemia OR hyperlipidemia OR hyperlipidaemia OR dyslipidemia OR dyslipidemia):ti,ab,kw

5	#1 OR #2 OR #3 OR #4

6	MeSH descriptor: [PCSK9 Inhibitors] explode all trees

7	("Proprotein Convertase 9"):ti,ab,kw

8	("proprotein convertase subtilisin kexin type 9"):ti,ab,kw

9	(alirocumab OR praluent OR REGN727 OR SAR236553):ti,ab,kw

10	(evolocumab OR repatha OR AMG145):ti,ab,kw

11	(Inclisiran OR leqvio OR ALN60212 OR ALN-PCS OR ALN-PCSsc):ti,ab,kw

12	(tafolecimab OR sintbilo):ti,ab,kw

13	(recaticimab OR SHR-1209):ti,ab,kw

14 (ongericimab OR JS002):ti,ab,kw

15 #6 OR #7 OR #8 OR #9 OR #10 OR #11 OR #12 OR #13 OR #14

16 MeSH descriptor: [Randomized Controlled Trial] explode all trees

17 MeSH descriptor: [Controlled Clinical Trial] explode all trees

18 (randomized OR randomised OR randomization OR randomly OR rct OR trial):ti,ab,kw

19 #16 OR #17 OR #18

20 (Animal*):ti,ab,kw

21 (Human*):ti,ab,kw

22 #20 NOT #21

23 #19 NOT #22

24 #5 AND #15 AND #23

# **Appendix 2: Characteristics of included studies**

## Table S2.1: Baseline of characteristics of included studies

| Study | Registration | Country/Region | Duration | Population | Treatments (N) | Age (I/C), yr | Male% (I/C) | On statin % (I/C) | Ezetimibe% (I/C) | Background lipid-lowering treatment |
| --- | --- | --- | --- | --- | --- | --- | --- | --- | --- | --- |
| Stiekema et al, 2019 ANITSCHKOW | NCT02729025 | Multinational (3) | 16w | Elevated Lp(a)/LDL-C | Evo: 65; PBO: 64 | 59.0 / 60.5 | 40.0 / 53.1 | 55.4 / 53.1 | 15.4 / 26.6 | Stable statin ± EZE |
| Rosenson et al, 2019 BANTING | NCT02739984 | Multinational (6) | 12w | Hypercholesterolaemia/dyslipidemia & T2DM | Evo: 280; PBO: 141 | 62.5 / 62.2 | 57.1 / 53.9 | 99.6 / 98.6 | NR | Max-tolerated statin |
| Boccara et al, 2020 BEIJERINCK | NCT02833844 | Multinational (4) | 48w | Hypercholesterolaemia/dyslipidemia & HIV | Evo: 307; PBO: 157 | 56.4 / 56.2 | 85.7 / 76.4 | 75.5 / 72.6 | 17.3 / 23.6 | Max-tolerated statin |
| Lorenzatti et al, 2019 BERSON | NCT02662569 | Multinational (10) | 14w | Hyperlipidaemia/Mixed dyslipidemia & T2DM | Evo: 657; PBO: 484 | 62.0 / 62.0 | 44.1 / 39.8 | 56.9 / 57.4 | NR | Atorvastatin 20 mg |
| Huo et al, 2023  CREDIT-1 | NCT04289285 | China | 52w | Non-familial hypercholesterolemia | Taf: 411; PBO: 203 | 57.8 / 57.0 | 66.4 / 65.1 | 99.25 / 99.05 | 9.4 / 8.9 | Mod/High statin ± EZE |
| Chai et al, 2023 CREDIT-2 | NCT04179669 | China | 32w | Hypercholesterolaemia (HeFH) | Taf: 100; PBO: 48 | 50.3 / 47.7 | 49.8 / 55.9 | 98.95 / 100 | 30.1 / 24.9 | Mod/High statin ± EZE |
| Qi et al, 2023 CREDIT-4 | NCT04709536 | China | 28w | Hypercholesterolaemia (High risk/HeFH) | Taf: 205; PBO: 98 | 56.9 / 56.8 | 71.7 / 63.3 | 100 / 100 | 11.2 /8.2 | Mod/High statin ± EZE |
| Blom et al, 2014 DESCARTES | NCT01516879 | Multinational (5) | 52w | Hyperlipidaemia & various CV risk | Evo: 599; PBO: 302 | 55.9 / 56.7 | 48.4 / 46.4 | 87.6 / 87.8 | 21.0 / 20.9 | Diet, Statin ± EZE |
| Koskinas et al, 2019 EVOPACS | NCT03287609 | Switzerland | 8w | dyslipidemia & ACS | Evo: 155; PBO: 153 | 60.5 / 61.0 | 83.0 / 80.0 | 20.0 / 23.0 | 4.0 / 6.0 | High-intensity statin |
| Sabatine et al, 2017 FOURIER | NCT01764633 | Multinational (48) | Median 2.2 years | dyslipidemia & ASCVD | Evo: 13784; PBO: 13780 | 62.5 / 62.5 | 75.4 / 75.5 | 99.7 / 99.8 | 5.3 / 5.2 | Mod/High statin ± EZE |
| Gao et al. 2021/2022 | NCT04851769 | China | 15 m | Hypercholesterolaemia & CAD | Ali: 30; SC: 31 | 61.3 / 61.3 | 66.7 / 74.2 | 26.7 / 32.3 | NR | Mod-intensity statin |
| Nicholls et al, 2016 GLAGOV | NCT01813422 | Multinational (32) | 78w | dyslipidemia & angiographic CAD | Evo: 484; PBO: 484 | 59.8 / 59.8 | 72.1 / 72.3 | 98.8 / 98.3 | 2.1 / 2.1 | Stable statin therapy |
| Hao et al, 2022 | NR | China | 3m | dyslipidemia & high-risk ACS | Evo: 68;  SC: 68 | 62.2 / 62.2 | 66.2 / 70.6 | NR | NR | High-intensity statin |
| Tan et al, 2023 HUATUO | NCT03433755 | China | 12w | Hypercholesterolaemia/Mixed dyslipidemia | Evo: 159; PBO: 82 | 61.0 / 58.6 | 64.2 / 74.4 | 100 / 100 | 17.7 / 20.7 | Stable statin ± LLT |
| Nicholls et al, 2022 HUYGENS | NCT03570697 | Multinational (7) | 52w | dyslipidemia & NSTEMI | Evo: 80; PBO: 81 | 60.9 / 60.2 | 75.0 / 67.9 | 93.8 / 96.3 | 1.3 / 2.5 | Maximally tolerated statin |
| Janik et al, 2021 | NR | Multinational (9) | 106w | Hypercholesterolaemia (HeFH or high risk) | Ali: 1087; PBO: 1084 | 63.0 / 63.0 | 58.8 / 57.7 | 90.1 / 90.8 | NR | Max-tolerated statin |
| Ji et al, 2023 | NR | China | 6m | Dyslipidemia & NSTE-ACS | Evo: 55;  SC: 55 | 60.5 / 61.2 | 69.1 / 65.4 | NR | NR | Atorvastatin 20 mg |
| Robinson et al, 2014 LAPLACE-2 | NCT01763866 | Multinational (17) | 12w | Hypercholesterolaemia/Mixed dyslipidemia | Evo: 1117;  PBO: 558 | 59.6 / 60.8 | 56.0 / 50.7 | 100 / 100 | 0 | Mod/High statin |
| Giugliano et al, 2012 LAPLACE-TIMI 57 | NCT01380730 | Multinational (5) | 12w | Hypercholesterolemic | Evo: 474; PBO: 157 | 62.2 / 62.0 | 49.2 / 46.5 | 99.7 / 98.5 | 9.0 / 9.5 | Stable statin ± EZE |
| Lin et al, 2025 | NCT05325203 | China | 32w | Heterozygous familial hypercholesterolemia (HeFH) | Ong: 88; PBO: 45 | 48.1 / 47.0 | 38.7 / 51.0 | 100/ 100 | 69.3 / 48.7 | Mod-intensity statin |
| McKenney et al, 2012 | NCT01288443 | USA | 20w | Hypercholesterolaemia | Ali: 152; PBO: 31 | 57.4 / 53.3 | 46.8 / 51.6 | 100 / 100 | 0 | Stable Atorvastatin |
| Koren et al, 2014 MENDEL-2 | NCT01763827 | Multinational (9) | 12w | Hypercholesterolaemia | Evo: 306; PBO: 154 | 53.0 / 53.5 | 33.0 / 38.5 | 0 | 0 | None |
| Schwartz et al, 2018 ODYSSEY OUTCOME | NCT01666457 | Multinational (56) | Median 2.8 years | dyslipidemia & recent ACS | Ali: 9462; PBO: 9462 | 58.5 / 58.6 | 74.7 / 74.9 | 97.6 / 97.6 | 2.8 / 3.0 | High-intensity statins |
| Stroes et al, 2016 ODYSSEY CHOICE II | NCT01730053 | Multinational (10) | 32w | Hypercholesterolaemia (No statin) | Ali: 175; PBO: 58 | 63.3 / 63.1 | 55.2 / 53.4 | 0 | 59.8 / 60.3 | None |
| Roth et al, 2016 ODYSSEY CHOICE I | NCT01926782 | Multinational (8) | 56w | Hypercholesterolemic | Ali: 573; PBO: 230 | 61.2 / 60.5 | 52.3 / 59.6 | 50.2 / 50.0 | 11.3 / 14.6 | Max statin or No statin |
| Kereiakes et al, 2015 ODYSSEY COMBO I | NCT01644175 | USA | 60w | Hypercholesterolaemia & high CV risk | Ali: 209; PBO: 107 | 63.0 / 63.0 | 62.7 / 72.0 | 99.5 / 100 | 7.2 / 10.3 | Max-tolerated statin |
| Leiter et al, 2017 ODYSSEY DM-INSULIN | NCT02585778 | Multinational (10) | 32w | dyslipidemia & high CV risk | Ali: 345; PBO: 172 | 62.6 / 63.2 | 55.9 / 60.6 | 73.05 / 70.45 | 9.6 / 9.4 | maximally tolerated statin |
| Moriarty et al, 2016 ODYSSEY ESCAPE | NCT02326220 | Germany, USA | 18w | HeFH | Ali: 41; PBO: 21 | 59.5 / 57.0 | 63.4 / 47.6 | 46.3 / 61.9 | NR | Max statin ± LLT |
| Kastelein et al, 2015 ODYSSEY FH I | NCT01623115 | Multinational (14) | 78w | HeFH | Ali: 323; PBO: 163 | 52.1 / 51.7 | 55.7/ 57.7 | 100 / 100 | 56.0 / 59.5 | Stable maximally tolerated statin |
| Kastelein et al, 2015 ODYSSEY FH II | NCT01709500 | Multinational (10) | 78w | HeFH | Ali: 167; PBO: 82 | 53.2 / 53.2 | 51.5 / 54.9 | 100 / 100 | 67.1 / 64.6 | Stable maximally tolerated statin |
| Ginsberg et al, 2016 ODYSSEY HIGH FH | NCT01617655 | Multinational (5) | 86w | HeFH | Ali: 72; PBO: 35 | 49.8 / 52.1 | 48.6 / 62.9 | 100 / 100 | 19.4 / 34.3 | Stable maximally tolerated statin |
| Blom et al, 2020 ODYSSEY HoFH | NCT01617486 | Multinational (13) | 32w | HoFH | Ali: 45; PBO: 24 | 42.3 / 45.4 | 46.7 / 54.2 | 97.8 / 95.8 | 68.9 / 79.2 | Stable LLT |
| Teramoto et al, 2016 ODYSSEY JAPAN | NCT02107898 | Japan | 60w | HeFH | Ali: 144; PBO: 72 | 60.3 / 61.8 | 58.3 / 65.3 | 100 / 100 | NR | Stable maximally tolerated statin |
| Koh et al, 2018 ODYSSEY KT | NCT02289963 | South Korea, Taiwan | 32w | Hypercholesterolaemia & high CV risk | Ali: 97; PBO: 102 | 61.2 / 60.1 | 85.6 / 79.4 | 100 / 100 | 14.4 / 11.8 | Stable maximally tolerated statin |
| Robinson et al, 2015 ODYSSEY LONG TERM | NCT01507831 | Multinational (29) | 86w | Hypercholesterolaemia (HeFH/High risk) | Ali: 1553; PBO: 788 | 60.4 / 60.6 | 63.3 / 60.2 | 100 / 100 | 13.9 / 15.0 | Stable maximally tolerated statin |
| Teramoto et al, 2019 ODYSSEY NIPPON | NCT02584504 | Japan | 64w | Japanese hypercholesterolemia | Ali: 107; PBO: 56 | 63.1 / 64.6 | 61.7 / 66.1 | 34.6 / 33.9 | 19.6 / 19.6 | Low-dose statin/diet |
| Bays et al, 2015 ODYSSEY OPTIONS I | NCT01730040 | Multinational (9) | 32w | Hypercholesterolaemia & high CVD risk | Ali: 104; SC: 149 | 63.2 / 64.8 / 61.2 | 62.0 / 63.3 / 69.7 | 100 / 100 / 100 | 0 | Stable statin |
| Farnier et al, 2016 ODYSSEY OPTIONS II | NCT01730053 | Multinational (8) | 32w | Hypercholesterolaemia & high CV risk | Ali: 103; SC: 101 | 60.0 / 61.8 / 61.05 | 57.6 / 56.4 / 70.25 | 100 / 100 / 100 | 0 | Stable statin |
| Ako et al, 2019 ODYSSEY J-IVUS | NCT02984982 | Japan | 36w | Hypercholesterolaemia & recent ACS | Ali: 104; SC: 102 | 61.8 / 60.5 | 79.6 / 80.9 | 100 / 100 | 7.5 / 7.9 | Stable statin |
| Ray et al, 2017 ORION-1 | NCT02597127 | Multinational (5) | 32w | Hypercholesterolaemia & high CV risk | Inc: 370; PBO: 127 | 63.6 / 62.4 | 67.0 / 59.1 | 72.4 / 73.5 | 30.0 / 30.5 | Max-tolerated statin |
| Raal et al, 2024  ORION-5 | NCT03851705 | Multinational (7) | 103w | HoFH | Inc: 37; PBO: 19 | 43.8 / 40.7 | 37.8 / 42.1 | 100 / 100 | 63.2 / 67.6 | Stable maximally tolerated statin |
| Raal et al, 2020 ORION-9 | NCT03397121 | Multinational (8) | 540d | HeFH | Inc: 242; PBO: 240 | 56.0 / 56.0 | 46.3 / 47.9 | 90.5 / 90.4 | 55.8 / 50.0 | Stable maximally tolerated statin |
| Ray et al, 2020 ORION-10 | NCT03399370 | USA | 540d | Hypercholesterolaemia & ASCVD | Inc: 781; PBO: 780 | 66.4 / 65.7 | 68.5 / 70.3 | 89.8 / 88.7 | 10.2 / 9.5 | Maximum tolerated dose of statin |
| Ray et al, 2020 ORION-11 | NCT03400800 | Multinational (7) | 540d | Hypercholesterolaemia & ASCVD | Inc: 810; PBO: 807 | 64.8 / 64.8 | 71.5 / 72.0 | 94.6 / 94.9 | 6.3 / 7.7 | Maximum tolerated dose of statin |
| Yamashita et al, 2024 ORION-15 | NCT04666298 | Japan | 360d | Japanese Hypercholesterolaemia (High risk) | Inc: 255; PBO: 57 | 63.5 / 63.8 | 74.2 / 68.4 | 90.0 / 87.7 | 41.3 / 36.8 | Maximally tolerated dose of statins |
| Huo et al, 2024 ORION-18 | NCT04765657 | China, South Korea, Singapore | 360d | Hypercholesterolaemia & ASCVD | Inc: 170; PBO: 174 | 58.8 / 60.1 | 76.5 / 73.0 | 98.8 / 96.6 | NR | Maximally tolerated dose of statins |
| Räber et al, 2022 PACMAN-AMI | NCT03067844 | Multinational (4) | 52w | dyslipidemia & recent ACS | Ali: 148; PBO: 152 | 58.4 / 58.6 | 83.8 / 78.3 | 11.5 / 13.2 | 0 / 0.7 | Rosuvastatin 20 mg |
| Xu et al, 2024 REMAIN-1 | NCT04849000 | China | 28w | Hypercholesterolaemia/Hyperlipidaemia | Rec: 468; PBO: 235 | 44.3 / 44.7 | 54.1 / 52.3 | 0 | 0 | None |
| Sun et al, 2024 REMAIN-2 | NCT04885218 | China | 52w | Non-familial Hypercholesterolaemia | Rec: 456; PBO: 233 | 56.0 / 55.6 | 63.6 / 66.1 | 100 / 100 | 10.7 / 12.0 | Mod/High statin ± EZE |
| Li et al, 2025 REMAIN-3 | NCT04844125 | China | 16w | HeFH | Rec: 95; PBO: 48 | 45.0 / 41.5 | 52.6 / 64.6 | 100 / 100 | 37.9 / 37.5 | Mod/High statin ± EZE |
| Roth et al, 2012 | NCT01288469 | USA | 16w | Primary Hypercholesterolemia | Ali: 61; PBO: 31 | 57.8 / 55.3 | 33.0 / 42.0 | 100 / 100 | 0 | Stable Atorvastatin |
| Raal et al, 2012 RUTHERFORD | NCT01375751 | Multinational (6) | 12w | HeFH | Evo: 111; PBO: 56 | 49.7 / 49.3 | 58.5 / 42.9 | 91.0 / 87.5 | 64.9 / 64.3 | Max-tolerated LLT |
| Raal et al, 2015 RUTHERFORD-2 | NCT01763918 | Multinational (12) | 14w | HeFH | Evo: 220; PBO: 109 | 52.3 / 49.0 | 59.0 / 55.0 | 100 / 100 | NR | Stable statin ± LLT |
| Shao et al, 2025 | NCT05621070 | China | 60w | Hypercholesterolaemia & statin intolerance | Ong: 92; PBO: 47 | 56.9 / 57.8 | 53.3 / 55.3 | 0 | 18.5 / 21.2 | None |
| Stein et al, 2012 | NCT01266876 | USA, Canada | 20w | HeFH | Ali: 62; PBO: 15 | 53.7 / 51.9 | 61.0 / 60.0 | 100 / 100 | 71.3 / 73.0 | Stable statin ± EZE |
| Uehara et al, 2024 Adage-Joto | UMIN000030588 | Japan | 9m | dyslipidemia & STEMI-ACS | ACSEvo: 29; SC: 23 | 59.6 / 60.0 | 75.8 / 69.5 | 100 / 100 | 7.0 / 26.0 | Intensive statin |
| Wang et al, 2024 | CTR20220027 | China | 20w | Hypercholesterolaemia/Mixed dyslipidemia | Ong: 169; PBO: 86 | 58.4 / 60.8 | 51.5 / 51.7 | 100 / 100 | 8.9 / 5.8 | Stable optimized LLT |
| Wang et al, 2024 | NCT04781114 | China | 60w | Hypercholesterolaemia/Mixed dyslipidemia | Ong: 537; PBO: 265 | 59.1 / 60.3 | 60.4 / 61.9 | 100 / 100 | 17.7 / 18.2 | Stable optimized LLT |
| Hirayama et al, 2014 YUKAWA | NCT01586520 | Japan | 12w | Japanese Hypercholesterolaemia (High risk) | Evo: 205; PBO: 102 | 61.9 / 60.6 | 61.3 / 70.6 | 100 / 100 | NR | Stable statin ± EZE |
| Kiyosue et al, 2016 YUKAWA-2 | NCT01953328 | Japan | 12w | Hyperlipidaemia/dyslipidemia | Evo: 202; PBO: 202 | 62.0 / 61.0 | 60.0 / 61.0 | 100 / 100 | NR | Atorvastatin 5/20 mg |
| Koren et al, 2012 MENDEL | NCT01375777 | Multinational (4) | 12w | Hypercholesterolaemia | Evo: 271; PBO: 90 | 50.1 / 51.25 | 31.0 / 37.5 | 0 | NR | None |
| Teramoto et al, 2016 | NCT01812707 | Japan | 20w | Japanese Hypercholesterolaemia | Ali: 75; PBO: 25 | 57.4 / 58.6 | 41.3 / 56.0 | 100 / 100 | NR | Atorvastatin 5/20 mg |

ACS: Acute Coronary Syndrome; Ali: Alirocumab; ASCVD: Atherosclerotic Cardiovascular Disease; ATV: Atorvastatin; CAD: Coronary Artery Disease; Dur. : Duration; Dyslip.: dyslipidemia; Evo: Evolocumab; EZE: Ezetimibe; HeFH: Heterozygous Familial Hypercholesterolaemia; HIV: Human Immunodeficiency Virus; HoFH: Homozygous Familial Hypercholesterolaemia; I/C: Intervention group / Control group; Inc: Inclisiran; LLT: Lipid-Lowering Therapy; Lp(a): Lipoprotein(a); m: Months; Mod/High: Moderate or high intensity; NR: Not reported; NSTE-ACS: Non-ST-elevation Acute Coronary Syndrome; NSTEMI: Non-ST-elevation Myocardial Infarction; Ong: Ongericimab; PBO: Placebo; PCI: Percutaneous Coronary Intervention; Rec: Recaticimab; SAMS: Statin-associated muscle symptoms; SC: Standard Care; STEMI-ACS: ST-elevation Myocardial Infarction Acute Coronary Syndrome; T2DM: Type 2 Diabetes Mellitus; Taf: Tafolecimab; w: Weeks; yr: Years

## References

[1] Stiekema LCA, Stroes ESG, Verweij SL, et al. Persistent arterial wall inflammation in patients with elevated lipoprotein(a) despite strong low-density lipoprotein cholesterol reduction by proprotein convertase subtilisin/kexin type 9 antibody treatment. Eur Heart J. 2018;40(33):2775–81. doi:10.1093/eurheartj/ehy862.
[2] Rosenson RS, Daviglus ML, Handelsman Y, et al. Efficacy and safety of evolocumab in individuals with type 2 diabetes mellitus: primary results of the randomised controlled BANTING study. Diabetologia. 2019;62(6):948–58. doi:10.1007/s00125-019-4856-7.
[3] Boccara F, Kumar PN, Caramelli B, et al. Evolocumab in HIV-infected patients with dyslipidemia: primary results of the randomized, double-blind BEIJERINCK study. J Am Coll Cardiol. 2020;75(20):2570–84. doi:10.1016/j.jacc.2020.03.025.
[4] Lorenzatti AJ, Eliaschewitz FG, Chen Y, et al. Randomised study of evolocumab in patients with type 2 diabetes and dyslipidemia on background statin: primary results of the BERSON clinical trial. Diabetes Obes Metab. 2019;21(6):1455–63. doi:10.1111/dom.13680.
[5] Huo Y, Chen B, Lian Q, et al. Tafolecimab in Chinese patients with non-familial hypercholesterolemia (CREDIT-1): a 48-week randomized, double-blind, placebo-controlled phase 3 trial. Lancet Reg Health West Pac. 2023;41:100907. doi:10.1016/j.lanwpc.2023.100907.
[6] Chai M, He Y, Zhao W, et al. Efficacy and safety of tafolecimab in Chinese patients with heterozygous familial hypercholesterolemia: a randomized, double-blind, placebo-controlled phase 3 trial (CREDIT-2). BMC Med. 2023;21(1):50. doi:10.1186/s12916-023-02797-8.
[7] Qi L, Liu D, Qu Y, et al. Tafolecimab in Chinese patients with hypercholesterolemia (CREDIT-4) a randomized, double-blind, placebo-controlled phase 3 trial. JACC Asia. 2023;3(4):636–45. doi:10.1016/j.jacasi.2023.04.011.
[8] Blom DJ, Hala T, Bolognese M, et al. A 52-week placebo-controlled trial of evolocumab in hyperlipidemia. N Engl J Med. 2014;370(19):1809–19. doi:10.1056/NEJMoa1316222.
[9] Koskinas KC, Windecker S, Pedrazzini G, et al. Evolocumab for early reduction of LDL cholesterol levels in patients with acute coronary syndromes (EVOPACS). J Am Coll Cardiol. 2019;74(20):2452–62. doi:10.1016/j.jacc.2019.08.010.
[10] Sabatine MS, Giugliano RP, Keech AC, et al. Evolocumab and clinical outcomes in patients with cardiovascular disease. N Engl J Med. 2017;376(18):1713–22. doi:10.1056/NEJMoa1615664.
[11] Gao F, Wang ZJ, Ma XT, Shen H, Yang LX, Zhou YJ. Effect of alirocumab on coronary plaque in patients with coronary artery disease assessed by optical coherence tomography. Lipids Health Dis. 2021;20(1):106. doi:10.1186/s12944-021-01528-3.
[12] Gao F, Li YP, Ma XT, Wang ZJ, Shi DM, Zhou YJ. Effect of alirocumab on coronary calcification in patients with coronary artery disease. Front Cardiovasc Med. 2022;9:907662. doi:10.3389/fcvm.2022.907662.
[13] Nicholls SJ, Puri R, Anderson T, et al. Effect of evolocumab on progression of coronary disease in statin-treated patients: the GLAGOV randomized clinical trial. JAMA. 2016;316(22):2373–84. doi:10.1001/jama.2016.16951.
[14] Hao Y, Yang YL, Wang YC, Li J. Effect of the early application of evolocumab on blood lipid profile and cardiovascular prognosis in patients with extremely high-risk acute coronary syndrome. Int Heart J. 2022;63(4):669–77. doi:10.1536/ihj.22-052.
[15] Tan H, Li W, Huang Z, et al. Efficacy and safety of evolocumab in Chinese patients with primary hypercholesterolemia and mixed dyslipidemia: 12-week primary results of the HUA TUO randomized clinical trial. Cardiol Ther. 2023;12:341–59. doi:10.1007/s40119-023-00304-x.
[16] Nicholls SJ, Kataoka Y, Nissen SE, et al. Effect of evolocumab on coronary plaque phenotype and burden in statin-treated patients following myocardial infarction. JACC Cardiovasc Imaging. 2022;15(7):1308–21. doi:10.1016/j.jcmg.2022.03.002.
[17] Janik MJ, Urbach DV, van Nieuwenhuizen E, et al. Alirocumab treatment and neurocognitive function according to the CANTAB scale in patients at increased cardiovascular risk: a prospective, randomized, placebo-controlled study. Atherosclerosis. 2021;331:20–7. doi:10.1016/j.atherosclerosis.2021.06.913.
[18] Ji J, Wei X, Chen W, Wan D, Han W, Liu H. Effects of early PCSK9 inhibitor application on inflammation levels and microcirculatory function after PCI in patients with NSTE-ACS. Am J Transl Res. 2023;15(5):3586–94.
[19] Robinson JG, Nedergaard BS, Rogers WJ, et al. Effect of evolocumab or ezetimibe added to moderate- or high-intensity statin therapy on LDL-C lowering in patients with hypercholesterolemia: the LAPLACE-2 randomized clinical trial. JAMA. 2014;311(18):1870–82. doi:10.1001/jama.2014.4030.
[20] Giugliano RP, Desai NR, Kohli P, et al. Efficacy, safety, and tolerability of a monoclonal antibody to proprotein convertase subtilisin/kexin type 9 in combination with a statin in patients with hypercholesterolaemia (LAPLACE-TIMI 57): a randomised, placebo-controlled, dose-ranging, phase 2 study. Lancet. 2012;380(9858):2007–17. doi:10.1016/S0140-6736(12)61770-X.
[21] Lin J, Ji Y, Wang G, et al. Efficacy and safety of ongericimab in Chinese patients with heterozygous familial hypercholesterolemia: a randomized, double-blind, placebo-controlled phase 3 trial. Atherosclerosis. 2025;403:119120. doi:10.1016/j.atherosclerosis.2025.119120.
[22] McKenney JM, Koren MJ, Kereiakes DJ, Hanotin C, Ferrand AC, Stein EA. Safety and efficacy of a monoclonal antibody to proprotein convertase subtilisin/kexin type 9 serine protease, SAR236553/REGN727, in patients with primary hypercholesterolemia receiving ongoing stable atorvastatin therapy. J Am Coll Cardiol. 2012;59(25):2344–53. doi:10.1016/j.jacc.2012.03.007.
[23] Koren MJ, Lundqvist P, Bolognese M, et al. Anti-PCSK9 monotherapy for hypercholesterolemia: the MENDEL-2 randomized, controlled phase III clinical trial of evolocumab. J Am Coll Cardiol. 2014;63(23):2531–40. doi:10.1016/j.jacc.2014.03.018.
[24] Schwartz GG, Steg PG, Szarek M, et al. Alirocumab and cardiovascular outcomes after acute coronary syndrome. N Engl J Med. 2018;379(22):2097–107. doi:10.1056/NEJMoa1801174.
[25] Stroes E, Guyton JR, Lepor N, et al. Efficacy and safety of alirocumab 150 mg every 4 weeks in patients with hypercholesterolemia not on statin therapy: the ODYSSEY CHOICE II study. J Am Heart Assoc. 2016;5(9):e003421. doi:10.1161/JAHA.116.003421.
[26] Roth EM, Moriarty PM, Bergeron J, et al. A phase III randomized trial evaluating alirocumab 300 mg every 4 weeks as monotherapy or add-on to statin: ODYSSEY CHOICE I. Atherosclerosis. 2016;254:254–62. doi:10.1016/j.atherosclerosis.2016.08.043.
[27] Kereiakes DJ, Robinson JG, Cannon CP, et al. Efficacy and safety of the proprotein convertase subtilisin/kexin type 9 inhibitor alirocumab among high cardiovascular risk patients on maximally tolerated statin therapy: the ODYSSEY COMBO I study. Am Heart J. 2015;169(6):906–15. doi:10.1016/j.ahj.2015.03.004.
[28] Leiter LA, Cariou B, Müller‐Wieland D, et al. Efficacy and safety of alirocumab in insulin‐treated individuals with type 1 or type 2 diabetes and high cardiovascular risk: the ODYSSEY DM‐INSULIN randomized trial. Diabetes Obes Metab. 2017;19(12):1781–92. doi:10.1111/dom.13114.
[29] Moriarty PM, Parhofer KG, Babirak SP, et al. Alirocumab in patients with heterozygous familial hypercholesterolaemia undergoing lipoprotein apheresis: the ODYSSEY ESCAPE trial. Eur Heart J. 2016;37(48):3588–95. doi:10.1093/eurheartj/ehw388.
[30] Kastelein JJP, Ginsberg HN, Langslet G, et al. ODYSSEY FH I and FH II: 78 week results with alirocumab treatment in 735 patients with heterozygous familial hypercholesterolaemia. Eur Heart J. 2015;36(43):2996–3003. doi:10.1093/eurheartj/ehv370.
[31] Ginsberg HN, Rader DJ, Raal FJ, et al. Efficacy and safety of alirocumab in patients with heterozygous familial hypercholesterolemia and LDL-C of 160 mg/dl or higher. Cardiovasc Drugs Ther. 2016;30(5):473–83. doi:10.1007/s10557-016-6685-y.
[32] Blom DJ, Harada-Shiba M, Rubba P, et al. Efficacy and safety of alirocumab in adults with homozygous familial hypercholesterolemia: the ODYSSEY HoFH trial. J Am Coll Cardiol. 2020;76(2):131–42. doi:10.1016/j.jacc.2020.05.027.
[33] Teramoto T, Kobayashi M, Tasaki H, et al. Efficacy and safety of alirocumab in Japanese patients with heterozygous familial hypercholesterolemia or at high cardiovascular risk with hypercholesterolemia not adequately controlled with statins: ODYSSEY JAPAN randomized controlled trial. Circ J. 2016;80(9):1980–7. doi:10.1253/circj.CJ-16-0387.
[34] Koh KK, Nam CW, Chao TH, et al. A randomized trial evaluating the efficacy and safety of alirocumab in South Korea and Taiwan (ODYSSEY KT). J Clin Lipidol. 2018;12(1):162–72. doi:10.1016/j.jacl.2017.09.007.
[35] Robinson JG, Farnier M, Krempf M, et al. Efficacy and safety of alirocumab in reducing lipids and cardiovascular events. N Engl J Med. 2015;372(16):1489–99. doi:10.1056/NEJMoa1501031.
[36] Teramoto T, Kiyosue A, Ishigaki Y, et al. Efficacy and safety of alirocumab 150 mg every 4 weeks in hypercholesterolemic patients on non-statin lipid-lowering therapy or lowest strength dose of statin: ODYSSEY NIPPON. J Cardiol. 2019;73(3):218–27. doi:10.1016/j.jjcc.2018.10.004.
[37] Bays H, Gaudet D, Weiss R, et al. Alirocumab as add-on to atorvastatin versus other lipid treatment strategies: ODYSSEY OPTIONS I randomized trial. J Clin Endocrinol Metab. 2015;100(8):3140–8. doi:10.1210/jc.2015-1520.
[38] Farnier M, Jones P, Severance R, et al. Efficacy and safety of adding alirocumab to rosuvastatin versus adding ezetimibe or doubling the rosuvastatin dose in high cardiovascular-risk patients: the ODYSSEY OPTIONS II randomized trial. Atherosclerosis. 2016;244:138–46. doi:10.1016/j.atherosclerosis.2015.11.010.
[39] Ako J, Hibi K, Tsujita K, et al. Effect of alirocumab on coronary atheroma volume in Japanese patients with acute coronary syndrome: the ODYSSEY J-IVUS trial. Circ J. 2019;83(10):2025–33. doi:10.1253/circj.CJ-19-0412.
[40] Ray KK, Landmesser U, Leiter LA, et al. Inclisiran in patients at high cardiovascular risk with elevated LDL cholesterol. N Engl J Med. 2017;376(15):1430–40. doi:10.1056/NEJMoa1615758.
[41] Raal F, Durst R, Bi R, et al. Efficacy, safety, and tolerability of inclisiran in patients with homozygous familial hypercholesterolemia: results from the ORION-5 randomized clinical trial. Circulation. 2023;149(5):354–62. doi:10.1161/CIRCULATIONAHA.122.063460.
[42] Raal FJ, Kallend D, Ray KK, et al. Inclisiran for the treatment of heterozygous familial hypercholesterolemia. N Engl J Med. 2020;382(16):1520–30. doi:10.1056/NEJMoa1913805.
[43] Ray KK, Wright RS, Kallend D, et al. Two phase 3 trials of inclisiran in patients with elevated LDL cholesterol. N Engl J Med. 2020;382(16):1507–19. doi:10.1056/NEJMoa1912387.
[44] Yamashita S, Kiyosue A, Maheux P, et al. Efficacy, safety, and pharmacokinetics of inclisiran in Japanese patients: results from ORION-15. J Atheroscler Thromb. 2024;31(6):780–94. doi:10.5551/jat.64454.
[45] Huo Y, Lesogor A, Lee CW, et al. Efficacy and safety of inclisiran in Asian patients: results from ORION-18. JACC Asia. 2024;4(2):123–34. doi:10.1016/j.jacasi.2023.09.006.
[46] Räber L, Ueki Y, Otsuka T, et al. Effect of alirocumab added to high-intensity statin therapy on coronary atherosclerosis in patients with acute myocardial infarction: the PACMAN-AMI randomized clinical trial. JAMA. 2022;327(18):1771–81. doi:10.1001/jama.2022.5218.
[47] Xu M, Wang Z, Zhang Y, et al. Recaticimab monotherapy for nonfamilial hypercholesterolemia and mixed hyperlipemia: the phase 3 REMAIN-1 randomized trial. J Am Coll Cardiol. 2024;84(20):2026–36. doi:10.1016/j.jacc.2024.07.035.
[48] Sun Y, Lv Q, Guo Y, et al. Recaticimab as add-on therapy to statins for nonfamilial hypercholesterolemia. J Am Coll Cardiol. 2024;84(20):2037–47. doi:10.1016/j.jacc.2024.09.012.
[49] Li L, Zhou Y, Deng C, et al. Recaticimab in adult heterozygous familial hypercholesterolaemia (REMAIN-3): a multicentre, randomized, double-blind, placebo-controlled phase 3 study. Cardiovasc Res. 2025;121(12):1856–64. doi:10.1093/cvr/cvaf155.
[50] Roth EM, McKenney JM, Hanotin C, Asset G, Stein EA. Atorvastatin with or without an antibody to PCSK9 in primary hypercholesterolemia. N Engl J Med. 2012;367(20):1891–900. doi:10.1056/NEJMoa1201832.
[51] Raal F, Scott R, Somaratne R, et al. Low-density lipoprotein cholesterol-lowering effects of AMG 145, a monoclonal antibody to proprotein convertase subtilisin/kexin type 9 serine protease in patients with heterozygous familial hypercholesterolemia. Circulation. 2012;126(20):2408–17. doi:10.1161/CIRCULATIONAHA.112.144055.
[52] Raal FJ, Stein EA, Dufour R, et al. PCSK9 inhibition with evolocumab (AMG 145) in heterozygous familial hypercholesterolemia (RUTHERFORD-2): a randomised, double-blind, placebo-controlled trial. Lancet. 2015;385(9965):331–40. doi:10.1016/S0140-6736(14)61399-4.
[53] Shao C, Zhang S, Cheng Z, et al. Efficacy and safety of ongericimab in Chinese statin-intolerant patients with primary hypercholesterolemia or mixed dyslipidemia: a randomized, placebo-controlled phase 3 trial. Atherosclerosis. 2025;407:120408. doi:10.1016/j.atherosclerosis.2025.120408.
[54] Stein EA, Gipe D, Bergeron J, et al. Effect of a monoclonal antibody to PCSK9, REGN727/SAR236553, to reduce low-density lipoprotein cholesterol in patients with heterozygous familial hypercholesterolaemia on stable statin dose with or without ezetimibe therapy: a phase 2 randomised controlled trial. Lancet. 2012;380(9836):29–36. doi:10.1016/S0140-6736(12)60771-5.
[55] Uehara H, Kajiya T, Abe M, et al. Early and short-term use of PCSK9-inhibitors on coronary plaque stability in acute coronary syndrome. Eur Heart J Open. 2024;4(4):oeae055. doi:10.1093/ehjopen/oeae055.
[56] Zhao W, Cheng Z, Ji X, et al. Efficacy and safety of ongericimab given by prefilled syringe or autoinjector in primary hypercholesterolemia and mixed hyperlipidemia. Nutr Metab Cardiovasc Dis. 2024;34(9):2217–25. doi:10.1016/j.numecd.2024.04.011.
[57] Wang X, Qiu M, Cheng Z, et al. Efficacy and safety of ongericimab in Chinese patients with primary hypercholesterolemia and mixed dyslipidemia. J Am Heart Assoc. 2024;13(11):e033669. doi:10.1161/JAHA.123.033669.
[58] Hirayama A, Honarpour N, Yoshida M, et al. Effects of evolocumab (AMG 145), a monoclonal antibody to PCSK9, in hypercholesterolemic, statin-treated Japanese patients at high cardiovascular risk. Circ J. 2014;78(5):1073–82. doi:10.1253/circj.CJ-14-0130.
[59] Kiyosue A, Honarpour N, Kurtz C, et al. A phase 3 study of evolocumab (AMG 145) in statin-treated Japanese patients at high cardiovascular risk. Am J Cardiol. 2016;117(1):40–7. doi:10.1016/j.amjcard.2015.10.021.
[60] Koren MJ, Scott R, Kim JB, et al. Efficacy, safety, and tolerability of a monoclonal antibody to proprotein convertase subtilisin/kexin type 9 as monotherapy in patients with hypercholesterolaemia (MENDEL): a randomised, double-blind, placebo-controlled, phase 2 study. Lancet. 2012;380(9858):1995–2006. doi:10.1016/S0140-6736(12)61771-1.
[61] Teramoto T, Kobayashi M, Uno K, et al. Efficacy and safety of alirocumab in Japanese subjects (phase 1 and 2 studies). Am J Cardiol. 2016;118(1):56–63. doi:10.1016/j.amjcard.2016.04.011.

## Table S2.2: PCSK9 inhibitors list

| Drug | Synonyms | Drug type | Company | Highest phase | Drug approved country/region | ATC | Note |
| --- | --- | --- | --- | --- | --- | --- | --- |
| Alirocumab | Praluent; REGN727;SAR236553 | Monoclonal antibody | Sanofi / Regeneron | Approved | USA, EU, China, Japan, Canada | C10AX14 | Fully human monoclonal antibody against PCSK9 |
| Evolocumab | Repatha; AMG 145 | Monoclonal antibody | Amgen | Approved | USA, EU, China, Japan, Canada | C10AX13 | Fully human monoclonal antibody against PCSK9 |
| Inclisiran | Leqvio; ALN60212; ALN-PCS;ALN-PCSsc | siRNA | Novartis | Approved | USA, EU, China | C10AX16 | Small interfering RNA (siRNA) targeting PCSK9 mRNA |
| Ongericimab | JS002 | Monoclonal antibody | Junshi Biosciences | Approved | China | N/A | Fully human monoclonal antibody against PCSK9 |
| Recaticimab | SHR-1209 | Monoclonal antibody | Hengrui Medicine | Approved | China | N/A | Fully human monoclonal antibody against PCSK9 |
| Tafolecimab | SINTBILO | Monoclonal antibody | Innovent Biologics | Approved | China | N/A | Fully human monoclonal antibody against PCSK9 |

# **Appendix 3: List of data extracted from the included randomized clinical trials**

| **Variable category** | **Data extracted** |
| --- | --- |
| **Study characteristics** | First author, year of publication, trial name, trial registration (NCT number), country/region, and total duration. |
| **Patient characteristics** | Clinical population (diagnosis or inclusion criteria), sample size (N), mean/median age, and gender (male %). |
| **Baseline comorbidities** | Proportion of participants with hypertension (HTN) and diabetes mellitus (DM). |
| **Treatment details** | Intervention and comparator names, specific dosage, frequency of administration. |
| **Background therapy** | Stable background lipid-lowering treatments, including the use of statins and ezetimibe (%). |
| **Efficacy outcomes** | Percentage changes from baseline in lipid profiles: Lp(a), LDL-C, non-HDL-C, TG, TC, HDL-C, and ApoB. |
| **Safety outcomes** | Incidence of adverse events (AEs) and serious adverse events (SAEs). |

# **Appendix 4: Risk of bias of randomized clinical trials**

## Figure S4.1: Overall risk of bias presented as percentage of each risk of bias item across all included studies.

As percentage (intention-to treat)

|  | Low risk | some concerns | High risk |
| --- | --- | --- | --- |
| Randomization process | 100 | 0 | 0 |
| Deviations from intended interventions | 93.5 | 6.5 | 0 |
| Missing outcome data | 98.4 | 1.6 | 0 |
| Measurement of the outcome | 100 | 0 | 0 |
| Selection of the reported result | 100 | 0 | 0 |
| Overall | 93.5 | 6.5 | 0 |

Green = Low risk, Red = High risk, Yellow = Some concerns

## Table S4.2: Study level risk of bias assessment using Cochrane risk of bias tool 2.0 for assessing risk of bias of randomized clinical trials.

| Unique ID | Study ID | Randomization process | Deviations from intended interventions | Missing outcome data | Measurement of the outcome | Selection of the reported result | Overall |
| --- | --- | --- | --- | --- | --- | --- | --- |
| Stiekema et al, 2019 ANITSCHKOW | NCT02729025 | Low | Low | Low | Low | Low | Low |
| Rosenson et al, 2019 BANTING | NCT02739984 | Low | Low | Low | Low | Low | Low |
| Boccara et al, 2020 BEIJERINCK | NCT02833844 | Low | Low | Low | Low | Low | Low |
| Lorenzatti et al, 2019 BERSON | NCT02662569 | Low | Low | Low | Low | Low | Low |
| Huo et al, 2023  CREDIT-1 | NCT04289285 | Low | Low | Low | Low | Low | Low |
| Chai et al, 2023 CREDIT-2 | NCT04179669 | Low | Low | Low | Low | Low | Low |
| Qi et al, 2023 CREDIT-4 | NCT04709536 | Low | Low | Low | Low | Low | Low |
| Blom et al, 2014 DESCARTES | NCT01516879 | Low | Low | Low | Low | Low | Low |
| Koskinas et al, 2019 EVOPACS | NCT03287609 | Low | Low | Low | Low | Low | Low |
| Sabatine et al, 2017 FOURIER | NCT01764633 | Low | Low | Low | Low | Low | Low |
| Gao et al. 2021/2022 | NCT04851769 | Low | Some concerns | Low | Low | Low | Some concerns |
| Nicholls et al, 2016 GLAGOV | NCT01813422 | Low | Low | Low | Low | Low | Low |
| Hao et al, 2022 | NR | Low | Some concerns | Low | Low | Low | Some concerns |
| Tan et al, 2023 HUATUO | NCT03433755 | Low | Low | Low | Low | Low | Low |
| Nicholls et al, 2022 HUYGENS | NCT03570697 | Low | Low | Low | Low | Low | Low |
| Janik et al, 2021 | NR | Low | Low | Low | Low | Low | Low |
| Ji et al, 2023 | NR | Some concerns | Some concerns | Low | Low | Low | Some concerns |
| Robinson et al, 2014 LAPLACE-2 | NCT01763866 | Low | Low | Low | Low | Low | Low |
| Giugliano et al, 2012 LAPLACE-TIMI 57 | NCT01380730 | Low | Low | Low | Low | Low | Low |
| Lin et al, 2025 | NCT05325203 | Low | Low | Low | Low | Low | Low |
| McKenney et al, 2012 | NCT01288443 | Low | Low | Low | Low | Low | Low |
| Koren et al, 2014 MENDEL-2 | NCT01763827 | Low | Low | Low | Low | Low | Low |
| Schwartz et al, 2018 ODYSSEY OUTCOME | NCT01666457 | Low | Low | Low | Low | Low | Low |
| Stroes et al, 2016 ODYSSEY CHOICE II | NCT01730053 | Low | Low | Low | Low | Low | Low |
| Roth et al, 2016 ODYSSEY CHOICE I | NCT01926782 | Low | Low | Low | Low | Low | Low |
| Kereiakes et al, 2015 ODYSSEY COMBO I | NCT01644175 | Low | Low | Low | Low | Low | Low |
| Leiter et al, 2017 ODYSSEY DM-INSULIN | NCT02585778 | Low | Low | Low | Low | Low | Low |
| Moriarty et al, 2016 ODYSSEY ESCAPE | NCT02326220 | Low | Low | Low | Low | Low | Low |
| Kastelein et al, 2015 ODYSSEY FH I | NCT01623115 | Low | Low | Low | Low | Low | Low |
| Kastelein et al, 2015 ODYSSEY FH II | NCT01709500 | Low | Low | Low | Low | Low | Low |
| Ginsberg et al, 2016 ODYSSEY HIGH FH | NCT01617655 | Low | Low | Low | Low | Low | Low |
| Blom et al, 2020 ODYSSEY HoFH | NCT01617486 | Low | Low | Low | Low | Low | Low |
| Teramoto et al, 2016 ODYSSEY JAPAN | NCT02107898 | Low | Low | Low | Low | Low | Low |
| Koh et al, 2018 ODYSSEY KT | NCT02289963 | Low | Low | Low | Low | Low | Low |
| Robinson et al, 2015 ODYSSEY LONG TERM | NCT01507831 | Low | Low | Low | Low | Low | Low |
| Teramoto et al, 2019 ODYSSEY NIPPON | NCT02584504 | Low | Low | Low | Low | Low | Low |
| Bays et al, 2015 ODYSSEY OPTIONS I | NCT01730040 | Low | Low | Low | Low | Low | Low |
| Farnier et al, 2016 ODYSSEY OPTIONS II | NCT01730053 | Low | Low | Low | Low | Low | Low |
| Ako et al, 2019 ODYSSEY J-IVUS | NCT02984982 | Low | Low | Low | Low | Low | Low |
| Ray et al, 2017 ORION-1 | NCT02597127 | Low | Low | Low | Low | Low | Low |
| Raal et al, 2024  ORION-5 | NCT03851705 | Low | Low | Low | Low | Low | Low |
| Raal et al, 2020 ORION-9 | NCT03397121 | Low | Low | Low | Low | Low | Low |
| Ray et al, 2020 ORION-10 | NCT03399370 | Low | Low | Low | Low | Low | Low |
| Ray et al, 2020 ORION-11 | NCT03400800 | Low | Low | Low | Low | Low | Low |
| Yamashita et al, 2024 ORION-15 | NCT04666298 | Low | Low | Low | Low | Low | Low |
| Huo et al, 2024 ORION-18 | NCT04765657 | Low | Low | Low | Low | Low | Low |
| Räber et al, 2022 PACMAN-AMI | NCT03067844 | Low | Low | Low | Low | Low | Low |
| Xu et al, 2024 REMAIN-1 | NCT04849000 | Low | Low | Low | Low | Low | Low |
| Sun et al, 2024 REMAIN-2 | NCT04885218 | Low | Low | Low | Low | Low | Low |
| Li et al, 2025 REMAIN-3 | NCT04844125 | Low | Low | Low | Low | Low | Low |
| Roth et al, 2012 | NCT01288469 | Low | Low | Low | Low | Low | Low |
| Raal et al, 2012 RUTHERFORD | NCT01375751 | Low | Low | Low | Low | Low | Low |
| Raal et al, 2015 RUTHERFORD-2 | NCT01763918 | Low | Low | Low | Low | Low | Low |
| Shao et al, 2025 | NCT05621070 | Low | Low | Low | Low | Low | Low |
| Stein et al, 2012 | NCT01266876 | Low | Low | Low | Low | Low | Low |
| Uehara et al, 2024 Adage-Joto | UMIN000030588 | Low | Some concerns | Some concerns | Low | Low | Some concerns |
| Wang et al, 2024 | CTR20220027 | Low | Low | Low | Low | Low | Low |
| Wang et al, 2024 | NCT04781114 | Low | Low | Low | Low | Low | Low |
| Hirayama et al, 2014 YUKAWA | NCT01586520 | Low | Low | Low | Low | Low | Low |
| Kiyosue et al, 2016 YUKAWA-2 | NCT01953328 | Low | Low | Low | Low | Low | Low |
| Koren et al, 2012 MENDEL | NCT01375777 | Low | Low | Low | Low | Low | Low |
| Teramoto et al, 2016 | NCT01812707 | Low | Low | Low | Low | Low | Low |

# **Appendix 5: Evaluation of heterogeneity**

## Table S5.1: Network heterogeneity

| **Clinical outcome** | **τ^2^ network** |
| --- | --- |
| Lp(a) | 0.67 |
| LDL-C | 0.79 |
| non-HDL-C | 0.54 |
| TG | 0.19 |
| TC | 0.35 |
| HDL-C | 0.15 |
| ApoB | 0.55 |
| AE | 0.01 |
| SAE | 0.05 |

Abbreviations: Lp(a), Lipoprotein(a); LDL-C, Low-Density Lipoprotein Cholesterol; non-HDL-C, Non-High-Density Lipoprotein Cholesterol; TG, Triglycerides; TC, Total Cholesterol; HDL-C, High-Density Lipoprotein Cholesterol; ApoB, Apolipoprotein B; AE, Adverse Event; SAE, Serious Adverse Event.

# **Appendix 6: Network plots and forest plots of secondary outcomes**

## Figure S6.1: Network plot of the effect on non-HDL-C, and forest plot of network effect sizes for compared with placebo.

Figure S6.2: Network plot of the effect on HDL-C, and forest plot of network effect sizes for compared with placebo.

# **Appendix 7: SUCRA and cumulative probability plots**

## Figure S7.1: Cumulative ranking curve plots of PCSK9 inhibitors for Lp(a) in range network.

| Treatment | SUCRA | PrBest | MeanRank |
| --- | --- | --- | --- |
| Ongericimab | 96.2 | 77.6 | 1.2 |
| Tafolecimab | 86.5 | 22.4 | 1.8 |
| Recaticimab | 59.1 | 0.0 | 3.5 |
| Evolocumab | 54.2 | 0.0 | 3.7 |
| Inclisiran | 30.6 | 0.0 | 5.2 |
| Alirocumab | 23.4 | 0.0 | 5.6 |
| Placebo | 0.0 | 0.0 | 7.0 |

Abbreviations: SUCRA, surface under the cumulative ranking curve.

## Figure S7.2: Cumulative ranking curve plots of PCSK9 inhibitors for LDL-C in range network.

| Treatment | SUCRA | PrBest | MeanRank |
| --- | --- | --- | --- |
| Ongericimab | 98.1 | 89.2 | 1.1 |
| Tafolecimab | 78.7 | 10.5 | 2.3 |
| Evolocumab | 66.8 | 0.1 | 3.0 |
| Recaticimab | 49.5 | 0.2 | 4.0 |
| Alirocumab | 30.5 | 0.0 | 5.2 |
| Inclisiran | 26.3 | 0.0 | 5.4 |
| Placebo | 0.0 | 0.0 | 7.0 |

## Figure S7.3: Cumulative ranking curve plots of PCSK9 inhibitors for non-HDL-C in range network.

| Treatment | SUCRA | PrBest | MeanRank |
| --- | --- | --- | --- |
| Ongericimab | 91.7 | 50.0 | 1.5 |
| Tafolecimab | 91.5 | 50.0 | 1.5 |
| Evolocumab | 64.1 | 0.0 | 3.2 |
| Recaticimab | 51.1 | 0.0 | 3.9 |
| Alirocumab | 28.2 | 0.0 | 5.3 |
| Inclisiran | 23.3 | 0.0 | 5.6 |
| Placebo | 0.0 | 0.0 | 7.0 |

## Figure S7.4: Cumulative ranking curve plots of PCSK9 inhibitors for TG in range network.

| Treatment | SUCRA | PrBest | MeanRank |
| --- | --- | --- | --- |
| Tafolecimab | 95.6 | 81.5 | 1.2 |
| Ongericimab | 84.1 | 17.7 | 2.0 |
| Evolocumab | 68.8 | 0.7 | 2.9 |
| Alirocumab | 45.1 | 0.0 | 4.3 |
| Recaticimab | 30.0 | 0.0 | 5.2 |
| Inclisiran | 26.5 | 0.0 | 5.4 |
| Placebo | 0.0 | 0.0 | 7.0 |

## Figure S7.5: Cumulative ranking curve plots of PCSK9 inhibitors for TC in range network.

| Treatment | SUCRA | PrBest | MeanRank |
| --- | --- | --- | --- |
| Tafolecimab | 90.2 | 54.0 | 1.5 |
| Ongericimab | 88.9 | 46.0 | 1.5 |
| Evolocumab | 60.6 | 0.0 | 3.0 |
| Alirocumab | 33.6 | 0.0 | 4.3 |
| Inclisiran | 26.7 | 0.0 | 4.7 |
| Placebo | 0.0 | 0.0 | 6.0 |

Figure S7.6: Cumulative ranking curve plots of PCSK9 inhibitors for HDL-C in range network.

| Treatment | SUCRA | PrBest | MeanRank |
| --- | --- | --- | --- |
| Ongericimab | 99.1 | 96.1 | 1.0 |
| Evolocumab | 74.8 | 2.0 | 2.3 |
| Inclisiran | 48.1 | 0.3 | 3.6 |
| Alirocumab | 46.4 | 0.0 | 3.7 |
| Tafolecimab | 30.9 | 1.6 | 4.5 |
| Placebo | 0.7 | 0.0 | 6.0 |

### **Figure S7.7: Cumulative ranking curve plots of PCSK9 inhibitors for ApoB in range network.**

| Treatment | SUCRA | PrBest | MeanRank |
| --- | --- | --- | --- |
| Ongericimab | 91.8 | 52.5 | 1.5 |
| Tafolecimab | 91.4 | 47.5 | 1.5 |
| Evolocumab | 60.6 | 0.0 | 3.4 |
| Recaticimab | 55.3 | 0.0 | 3.7 |
| Alirocumab | 31.2 | 0.0 | 5.1 |
| Inclisiran | 19.7 | 0.0 | 5.8 |
| Placebo | 0.0 | 0.0 | 7.0 |

## Figure S7.8: Cumulative ranking curve plots of PCSK9 inhibitors for AE in range network.

| Treatment | SUCRA | PrBest | MeanRank |
| --- | --- | --- | --- |
| Ongericimab | 92.2 | 63.0 | 1.5 |
| Tafolecimab | 81.6 | 35.5 | 2.0 |
| Placebo | 51.9 | 0.5 | 3.9 |
| Evolocumab | 51.2 | 0.1 | 3.9 |
| Alirocumab | 31.2 | 0.1 | 5.2 |
| Inclisiran | 26.1 | 0.1 | 5.4 |
| Recaticimab | 15.8 | 0.7 | 6.0 |

## Figure S7.9: Cumulative ranking curve plots of PCSK9 inhibitors for SAE in range network.

| Treatment | SUCRA | PrBest | MeanRank |
| --- | --- | --- | --- |
| Inclisiran | 81.9 | 37.6 | 2.1 |
| Tafolecimab | 62.8 | 31.0 | 3.3 |
| Alirocumab | 55.9 | 4.4 | 3.7 |
| Placebo | 43.0 | 0.2 | 4.5 |
| Ongericimab | 39.7 | 11.2 | 4.5 |
| Recaticimab | 39.4 | 14.5 | 4.6 |
| Evolocumab | 27.2 | 1.0 | 5.3 |

# **Appendix 8: League table of Summary Estimates for PCSK9 inhibitors**

## Table S8.1: Lp(a)

| Alirocumab |  |  |  |  |  |  |
| --- | --- | --- | --- | --- | --- | --- |
| 5.48 (1.21,9.74) | Evolocumab |  |  |  |  |  |
| 1.20 (-5.25,7.65) | -4.27 (-10.64,2.09) | Inclisiran |  |  |  |  |
| 23.74 (13.66,33.83) | 18.27 (8.24,28.30) | 22.54 (11.41,33.67) | Ongericimab |  |  |  |
| 7.09 (-0.51,14.68) | 1.61 (-5.92,9.14) | 5.88 (-3.06,14.83) | -16.66 (-28.49,-4.82) | Recaticimab |  |  |
| 18.50 (8.41,28.60) | 13.03 (2.98,23.08) | 17.30 (6.15,28.45) | -5.24 (-18.82,8.34) | 11.42 (-0.44,23.27) | Tafolecimab |  |
| -22.62 (-25.71,-19.53) | -28.09 (-31.03,-25.16) | -23.82 (-29.47,-18.16) | -46.36 (-55.96,-36.77) | -29.70 (-36.64,-22.77) | -41.12 (-50.73,-31.51) | Placebo |

## Table S8.2: LDL-C

| Alirocumab |  |  |  |  |  |  |
| --- | --- | --- | --- | --- | --- | --- |
| 7.84 (3.02,12.65) | Evolocumab |  |  |  |  |  |
| -1.11 (-8.26,6.04) | -8.95 (-15.82,-2.08) | Inclisiran |  |  |  |  |
| 20.60 (11.16,30.05) | 12.77 (3.54,22.00) | 21.71 (11.07,32.35) | Ongericimab |  |  |  |
| 4.12 (-5.05,13.29) | -3.72 (-12.67,5.23) | 5.23 (-5.17,15.63) | -16.48 (-28.57,-4.40) | Recaticimab |  |  |
| 12.05 (0.86,23.24) | 4.22 (-6.80,15.23) | 13.16 (0.94,25.38) | -8.55 (-22.24,5.14) | 7.93 (-5.57,21.43) | Tafolecimab |  |
| -49.05 (-52.73,-45.36) | -56.88 (-59.98,-53.79) | -47.94 (-54.07,-41.81) | -69.65 (-78.35,-60.96) | -53.17 (-61.57,-44.77) | -61.10 (-71.67,-50.53) | Placebo |

## Table S8.3: non-HDL-C

| Alirocumab |  |  |  |  |  |  |
| --- | --- | --- | --- | --- | --- | --- |
| 8.75  (5.07,12.42) | Evolocumab |  |  |  |  |  |
| -1.10 (-6.40,4.19) | -9.85 (-15.09,-4.62) | Inclisiran |  |  |  |  |
| 20.28 (13.49,27.06) | 11.53 (4.79,18.27) | 21.38 (13.64,29.12) | Ongericimab |  |  |  |
| 5.46 (-0.95,11.88) | -3.28 (-9.65,3.08) | 6.57 (-0.85,13.99) | -14.81 (-23.36,-6.27) | Recaticimab |  |  |
| 20.17 (11.95,28.39) | 11.43 (3.24,19.62) | 21.28 (12.24,30.31) | -0.10 (-10.08,9.88) | 14.71 (4.98,24.44) | Tafolecimab |  |
| -43.16 (-45.81,-40.51) | -51.91 (-54.45,-49.37) | -42.05 (-46.63,-37.48) | -63.44 (-69.68,-57.19) | -48.62 (-54.46,-42.78) | -63.33 (-71.12,-55.55) | Placebo |

## Table S8.4: TG

| Alirocumab |  |  |  |  |  |  |
| --- | --- | --- | --- | --- | --- | --- |
| 3.32 (0.59,6.05) | Evolocumab |  |  |  |  |  |
| -2.30 (-5.97,1.37) | -5.62 (-9.32,-1.92) | Inclisiran |  |  |  |  |
| 7.74 (0.39,15.10) | 4.42 (-2.94,11.79) | 10.04 (2.28,17.81) | Ongericimab |  |  |  |
| -2.08 (-7.39,3.24) | -5.40 (-10.73,-0.06) | 0.23 (-5.65,6.10) | -9.82 (-18.49,-1.15) | Recaticimab |  |  |
| 13.93 (1.92,25.95) | 10.61 (-1.41,22.64) | 16.23 (3.96,28.51) | 6.19 (-7.63,20.01) | 16.01 (3.15,28.87) | Tafolecimab |  |
| -11.21 (-13.11,-9.31) | -14.53 (-16.49,-12.57) | -8.91 (-12.05,-5.76) | -18.95 (-26.05,-11.85) | -9.13 (-14.10,-4.17) | -25.14 (-37.00,-13.28) | Placebo |

## Table S8.5: TC

| Alirocumab |  |  |  |  |  |
| --- | --- | --- | --- | --- | --- |
| 4.59 (1.53,7.65) | Evolocumab |  |  |  |  |
| -0.97 (-5.32,3.38) | -5.56 (-9.79,-1.33) | Inclisiran |  |  |  |
| 14.80 (9.32,20.27) | 10.21 (4.83,15.59) | 15.77 (9.57,21.97) | Ongericimab |  |  |
| 15.61 (3.16,28.06) | 11.02 (-1.39,23.43) | 16.58 (3.79,29.37) | 0.81 (-12.40,14.02) | Tafolecimab |  |
| -30.59 (-32.86,-28.32) | -35.18 (-37.23,-33.13) | -29.62 (-33.32,-25.91) | -45.39 (-50.37,-40.41) | -46.20 (-58.44,-33.96) | Placebo |

## Table S8.6: HDL-C

| Alirocumab |  |  |  |  |  |
| --- | --- | --- | --- | --- | --- |
| -1.02 (-2.24,0.19) | Evolocumab |  |  |  |  |
| -0.00 (-1.78,1.77) | 1.02 (-0.75,2.79) | Inclisiran |  |  |  |
| -3.75 (-6.42,-1.08) | -2.73 (-5.41,-0.04) | -3.75 (-6.72,-0.77) | Ongericimab |  |  |
| 1.75 (-2.73,6.23) | 2.77 (-1.71,7.25) | 1.75 (-2.91,6.42) | 5.50 (0.42,10.58) | Tafolecimab |  |
| 5.79 (4.94,6.64) | 6.81 (5.95,7.67) | 5.79 (4.24,7.35) | 9.54 (7.00,12.08) | 4.04 (-0.36,8.44) | Placebo |

## Table S8.7: ApoB

| Alirocumab |  |  |  |  |  |  |
| --- | --- | --- | --- | --- | --- | --- |
| 6.95 (3.35,10.54) | Evolocumab |  |  |  |  |  |
| -2.55 (-7.78,2.68) | -9.49 (-14.64,-4.34) | Inclisiran |  |  |  |  |
| 19.42 (12.76,26.07) | 12.47 (5.88,19.06) | 21.96 (14.36,29.57) | Ongericimab |  |  |  |
| 5.87 (-0.49,12.23) | -1.08 (-7.37,5.22) | 8.42 (1.07,15.77) | -13.55 (-21.97,-5.12) | Recaticimab |  |  |
| 19.23 (11.00,27.46) | 12.29 (4.11,20.47) | 21.78 (12.76,30.80) | -0.18 (-10.10,9.73) | 13.36 (3.65,23.08) | Tafolecimab |  |
| -40.09 (-42.72,-37.47) | -47.04 (-49.50,-44.58) | -37.55 (-42.07,-33.02) | -59.51 (-65.63,-53.39) | -45.96 (-51.76,-40.17) | -59.33 (-67.13,-51.53) | Placebo |

## Table S8.8: AEs

| Alirocumab |  |  |  |  |  |  |
| --- | --- | --- | --- | --- | --- | --- |
| 1.06 (0.90,1.26) | Evolocumab |  |  |  |  |  |
| 0.97 (0.78,1.22) | 0.92 (0.74,1.14) | Inclisiran |  |  |  |  |
| 1.43 (1.02,2.00) | 1.35 (0.97,1.87) | 1.47 (1.02,2.10) | Ongericimab |  |  |  |
| 0.89 (0.62,1.27) | 0.84 (0.59,1.19) | 0.91 (0.62,1.34) | 0.62 (0.39,0.98) | Recaticimab |  |  |
| 1.31 (0.91,1.89) | 1.23 (0.86,1.77) | 1.34 (0.91,1.98) | 0.92 (0.58,1.45) | 1.47 (0.91,2.38) | Tafolecimab |  |
| 1.06 (0.93,1.21) | 1.00 (0.89,1.12) | 1.09 (0.91,1.31) | 0.74 (0.55,1.01) | 1.20 (0.85,1.67) | 0.81 (0.58,1.14) | Placebo |

## Table S8.9: SAEs

| Alirocumab |  |  |  |  |  |  |
| --- | --- | --- | --- | --- | --- | --- |
| 0.83 (0.56,1.24) | Evolocumab |  |  |  |  |  |
| 1.24 (0.81,1.91) | 1.49 (0.92,2.42) | Inclisiran |  |  |  |  |
| 0.89 (0.43,1.84) | 1.07 (0.50,2.28) | 0.72 (0.33,1.56) | Ongericimab |  |  |  |
| 0.87 (0.37,2.09) | 1.05 (0.43,2.57) | 0.70 (0.28,1.75) | 0.98 (0.33,2.89) | Recaticimab |  |  |
| 1.10 (0.51,2.41) | 1.32 (0.59,2.97) | 0.89 (0.39,2.03) | 1.24 (0.45,3.40) | 1.26 (0.41,3.87) | Tafolecimab |  |
| 0.93 (0.74,1.19) | 1.12 (0.81,1.55) | 0.75 (0.52,1.08) | 1.05 (0.53,2.08) | 1.07 (0.46,2.47) | 0.85 (0.40,1.78) | Placebo |

# **Appendix 9: CINeMA Assessment**

We use the CINeMA framework to evidence certainty, assessing it for each network estimate based on the following criteria:

**A: Within study bias:** We classified the overall risk of bias for each study as low risk of bias, the risk of bias as moderate when none of the four assessed risk of bias items were rated as high risk, and the risk of bias as high when one or both items were rated as high risk. See Appendix 4 for the bias assessment. The

risk of bias for a pairwise comparison of each drug is shown in figure S9.1-9.6.

## Figure S9.1: Risk of bias contribution by intervention group in Lp(a).

## Figure S9.2: Overall risk of bias by treatment comparison in Lp(a).

## Figure S9.3: Risk of bias contribution by intervention group in LDL-C.

## Figure S9.4: Overall risk of bias by treatment comparison in LDL-C.

## Table S9.1: Transitivity assessment.

|  | Baseline variable (Mean ± SD) | | |
| --- | --- | --- | --- |
| Intervention | Age (Year) | Lp(a) (nmol/L / mg/dL) | LDL-C (mmol/L) |
| Alirocumab | 58.5 ± 9.8 | 57.93 ± 47.91 nmol/L | 2.92 ± 1.06 |
|  |  | 27.93 ± 37.91 mg/dL |  |
| Evolocumab | 61.23 ± 9.9 | 74.00 ± 61.05 nmol/L | 3.05 ± 1.04 |
|  |  | 24.70 ± 36.50 mg/dL |  |
| Inclisiran | 63.51 ± 10.45 | 79.69 ± 115.74 nmol/L | 3.02 ± 1.35 |
| Ongericimab | 57.61 ± 10.79 | 59.98 ± 80.18 nmol/L | 2.98 ± 1.10 |
| Recaticimab | 50.11 ± 11.05 | 113.18 ± 239.66 nmol/L | 3.29 ± 0.81 |
| Tafolecimab | 56.49 ± 9.68 | 27.50 ± 30.50 mg/dL | 3.11 ± 0.99 |

**Imprecision:** We use the CINeMA website to grade the accuracy of each comparison.

**Heterogeneity:** We assessed the degree of worry by comparing clinical reasoning based on 95% confidence intervals (CIs) while applying the same clinical

reasoning framework as for inaccuracy. In particular, we judged the consistency of our findings based on the confidence and prediction intervals associated with

clinically important effect sizes. And we used the same thresholds of clinical significance as described above and followed the recommendations automatically

provided by CINeMA (https://cinema.ispm.unibe.ch/).

**Inconsistency:** For inconsistency, we looked at the results for node splitting (Appendix 5).

## Table S9.2: CINeMA Results of Lp(a).

| Comparison | Within-study bias | Reporting bias | Indirectness | Imprecision | Heterogeneity | Incoherence | Confidence rating |
| --- | --- | --- | --- | --- | --- | --- | --- |
| Alirocumab:Placebo | No concerns | Low risk | No concerns | No concerns | No concerns | Major concerns | Low |
| Evolocumab:Placebo | No concerns | Low risk | No concerns | No concerns | No concerns | Major concerns | Low |
| Inclisiran:Placebo | No concerns | Low risk | No concerns | No concerns | No concerns | Major concerns | Low |
| Ongericimab:Placebo | No concerns | Low risk | No concerns | No concerns | No concerns | Major concerns | Low |
| Placebo:Recaticimab | No concerns | Low risk | No concerns | No concerns | No concerns | Major concerns | Low |
| Placebo:Tafolecimab | No concerns | Low risk | No concerns | No concerns | No concerns | Major concerns | Low |
| Alirocumab:Evolocumab | No concerns | Low risk | No concerns | No concerns | Major concerns | Major concerns | Very low |
| Alirocumab:Inclisiran | No concerns | Low risk | No concerns | Major concerns | No concerns | Major concerns | Very low |
| Alirocumab:Ongericimab | No concerns | Low risk | No concerns | No concerns | No concerns | Major concerns | Low |
| Alirocumab:Recaticimab | No concerns | Low risk | No concerns | No concerns | Major concerns | Major concerns | Very low |
| Alirocumab:Tafolecimab | No concerns | Low risk | No concerns | No concerns | No concerns | Major concerns | Low |
| Evolocumab:Inclisiran | No concerns | Low risk | No concerns | Major concerns | No concerns | Major concerns | Very low |
| Evolocumab:Ongericimab | No concerns | Low risk | No concerns | No concerns | No concerns | Major concerns | Low |
| Evolocumab:Recaticimab | No concerns | Low risk | No concerns | Major concerns | No concerns | Major concerns | Very low |
| Evolocumab:Tafolecimab | No concerns | Low risk | No concerns | No concerns | Major concerns | Major concerns | Very low |
| Inclisiran:Ongericimab | No concerns | Low risk | No concerns | No concerns | No concerns | Major concerns | Low |
| Inclisiran:Recaticimab | No concerns | Low risk | No concerns | Major concerns | No concerns | Major concerns | Very low |
| Inclisiran:Tafolecimab | No concerns | Low risk | No concerns | No concerns | No concerns | Major concerns | Low |
| Ongericimab:Recaticimab | No concerns | Low risk | No concerns | No concerns | No concerns | Major concerns | Low |
| Ongericimab:Tafolecimab | No concerns | Low risk | No concerns | Major concerns | No concerns | Major concerns | Very low |
| Recaticimab:Tafolecimab | No concerns | Low risk | No concerns | No concerns | Major concerns | Major concerns | Very low |

## Table S9.3: CINeMA Results of LDL-C.

| Comparison | Within-study bias | Reporting bias | Indirectness | Imprecision | Heterogeneity | Incoherence | Confidence rating |
| --- | --- | --- | --- | --- | --- | --- | --- |
| Alirocumab:Placebo | No concerns | Low risk | No concerns | No concerns | No concerns | Major concerns | Low |
| Evolocumab:Placebo | No concerns | Low risk | No concerns | No concerns | No concerns | Major concerns | Low |
| Inclisiran:Placebo | No concerns | Low risk | No concerns | No concerns | No concerns | Major concerns | Low |
| Ongericimab:Placebo | No concerns | Low risk | No concerns | No concerns | No concerns | Major concerns | Low |
| Placebo:Recaticimab | No concerns | Low risk | No concerns | No concerns | No concerns | Major concerns | Low |
| Placebo:Tafolecimab | No concerns | Low risk | No concerns | No concerns | No concerns | Major concerns | Low |
| Alirocumab:Evolocumab | No concerns | Low risk | No concerns | No concerns | Major concerns | Major concerns | Very low |
| Alirocumab:Inclisiran | No concerns | Low risk | No concerns | Major concerns | No concerns | Major concerns | Very low |
| Alirocumab:Ongericimab | No concerns | Low risk | No concerns | No concerns | No concerns | Major concerns | Low |
| Alirocumab:Recaticimab | No concerns | Low risk | No concerns | Major concerns | No concerns | Major concerns | Very low |
| Alirocumab:Tafolecimab | No concerns | Low risk | No concerns | No concerns | Major concerns | Major concerns | Very low |
| Evolocumab:Inclisiran | No concerns | Low risk | No concerns | No concerns | Major concerns | Major concerns | Very low |
| Evolocumab:Ongericimab | No concerns | Low risk | No concerns | No concerns | Major concerns | Major concerns | Very low |
| Evolocumab:Recaticimab | No concerns | Low risk | No concerns | Major concerns | No concerns | Major concerns | Very low |
| Evolocumab:Tafolecimab | No concerns | Low risk | No concerns | Major concerns | No concerns | Major concerns | Very low |
| Inclisiran:Ongericimab | No concerns | Low risk | No concerns | No concerns | No concerns | Major concerns | Low |
| Inclisiran:Recaticimab | No concerns | Low risk | No concerns | Major concerns | No concerns | Major concerns | Very low |
| Inclisiran:Tafolecimab | No concerns | Low risk | No concerns | No concerns | Major concerns | Major concerns | Very low |
| Ongericimab:Recaticimab | No concerns | Low risk | No concerns | No concerns | Major concerns | Major concerns | Very low |
| Ongericimab:Tafolecimab | No concerns | Low risk | No concerns | Major concerns | No concerns | Major concerns | Very low |
| Recaticimab:Tafolecimab | No concerns | Low risk | No concerns | Major concerns | No concerns | Major concerns | Very low |

# **Appendix 10: Funnel plots**

## Figure S10.1: Funnel plot of Lpa.

Figure S10.2: Funnel plot of LDL-C.

## Figure S10.3: Funnel plot of non-HDL-C.

## Figure S10.4: Funnel plot of TG.

## Figure S10.5: Funnel plot of TC.

## Figure S10.6: Funnel plot of HDL-C.

## Figure S10.7: Funnel plot of ApoB.

## Figure S10.8: Funnel plot of AE.

## Figure S10.9: Funnel plot of SAE.

# **Appendix 11: Comprehensive comparisons of different PCSK9 inhibitors at various doses and dosing regimens**

## Figure S11.1: Lp(a)

# **Appendix 12: Subgroup analysis of PCSK9 inhibitors stratified by Lp(a) efficacy analysis time points**

## Figure S12.1: Early response (≤12 weeks)

## Figure S12.2: Mid-term maintenance (13–26 weeks)

## Figure S12.3: Long-term durability (>26 weeks)

# **Appendix 13: Subgroup analysis of PCSK9 inhibitors stratified by background statin therapy**

## Figure S13.1: With background statins

## Figure S13.2: Without background statins

# **Appendix 14: Comparison of the effects of PCSK9 inhibitors on Lp(a) levels between East Asian and Global populations**

## Figure S14.1: East Asian

East Asian: China, Japan, South Korea, and Singapore.

## Figure S14.2: Global

# **Appendix 15: Sensitivity analysis**

## Table S15.1 Sensitivity analysis of treatment effects on Lp(a) according to the reported measurement unit (mg/dL vs. nmol/L)

| PCSK9 inhibitors | Lp(a) | | |
| --- | --- | --- | --- |
|  | Main estimate | Sensitivity analysis (mg/dL) | Sensitivity analysis (nmol/L) |
| Ongericimab | -46.36 (-55.96,-36.77) | NA | -45.93 (-54.59,-37.26) |
| Tafolecimab | -41.12 (-50.73,-31.51) | -41.05 (-51.76,-30.33) | NA |
| Recaticimab | -29.70 (-36.64,-22.77) | NA | -29.66 (-35.75,-23.58) |
| Evolocumab | -28.09 (-31.03,-25.16) | -34.93 (-47.12,-22.74) | -27.49 (-30.18,-24.79) |
| Inclisiran | -23.82 (-29.47,-18.16) | NA | -23.63 (-28.66,-18.61) |
| Alirocumab | -22.62 (-25.71,-19.53) | -22.95 (-26.53,-19.36) | -19.25 (-40.48,1.99) |

## Figure S15.1 Sensitivity analysis of treatment effects on Lp(a) restricted to standard approved doses

# **Appendix 16: Specific AEs**

| Study | Injectionsite reactions | Muscle-related events | Hepatic effects (ALT/AST) | Neurocognitive events | Diabetes/Glucose control | Nasopharyngitis | Upper respiratory tract infection | Urinary tract infection | Headache |
| --- | --- | --- | --- | --- | --- | --- | --- | --- | --- |
| Stiekema 2019 ANITSCHKOW | NA | NA | NA | NA | NA | NA | NA | NA | NA |
| Rosenson et al. 2019 BANTING | NA | NA | 1 (0.4%) / 1 (0.7%) | NA | 8 (2.9%) / 5 (3.5%) | NA | 5 (1.8%) / 4 (2.8%) | 6 (2.1%) / 2 (1.4%) | 6 (2.1%) / 3 (2.1%) |
| Boccara et al. 2020 BEIJERINCK | 7 (2.3%) / 8 (5.1%) | NA | 1 (0.4%) / 0 (0.0%) | NA | NA | 10 (3.3%) / 2 (1.3%) | 7 (2.3%) / 4 (2.5%) | NA | NA |
| Lorenzatti et al. 2019 BERSON | NA | NA | 1 (0.2%) / 2 (0.6%) | NA | 38 (5.8%) / 8 (2.4%) | 20 (3.0%) / 12 (3.7%) | 23 (3.5%) / 11 (3.4%) | NA | 8 (1.2%) / 3 (0.9%) |
| HUO et al. 2023 CREDIT-1 | 30 (7.3%) / 5 (2.5%) | 19 (4.6%) / 5 (2.5%) | 10 (2.4%) / 3 (1.5%) | NA | 26 (6.3%) / 13 (6.4%) | NA | 46 (11.2%) / 21 (10.3%) | 45 (10.9%) / 21 (10.3%) | NA |
| Chai et al. 2023 CREDIT-2 | 3 (3.0%) / 0 (0.0%) | 5 (5.0%) / 3 (6.3%) | 2 (3.8%) / 0 (0%) | NA | NA | NA | 13 (13.0%) / 4 (8.3%) | NA | NA |
| Qi et al. 2023 CREDIT-4 | 2 (1.0%) / 1 (1.0%) | 2 (1.0%) / 3 (3.1%) | 3 (1.5%) / 2 (2.0%) | NA | NA | NA | 6 (2.9%) / 3 (3.1%) | 12 (5.9%) / 4 (4.1%) | NA |
| Blom et al. 2014 DESCARTES | 34 (5.7%) / 15 (5.0%) | 24 (4.0%) / 9 (3.0%) | NA | NA | NA | 63 (10.5%) / 29 (9.6%) | 56 (9.3%) / 19 (6.3%) | 27 (4.5%) / 11 (3.6%) | 24 (4.0%) / 11 (3.6%) |
| Koskinas et al. 2019 EVOPACS | 5 (3.2%) / 3 (2.0%) | 9 (5.8%) / 5 (3.3%) | 2 (1.3%) / 2 (1.3%) | 1 (0.6%) / 0 (0.0%) | NA | 4 (2.6%) / 3 (2.0%) | NA | NA | NA |
| Sabatine et al. 2017 FOURIER | 296 (2.1%) / 219 (1.6%) | 682 (5.0%) / 656 (4.8%) | 240 (1.8%) / 242 (1.8%) | 217 (1.6%) / 202 (1.5%) | 677 (8.1%) / 644 (7.7%) | NA | NA | NA | NA |
| Gao et al. 2021  NCT04851769 | 2 (6.7%) / 0 (0%) | NA | NA | NA | NA | 2 (6.7%) / 1 (3.2%) | NA | NA | NA |
| Nicholls 2016 GLAGOV | 2 (0.4%) / 0 (0%) | 34 (7.0%) / 28 (5.8%) | 2 (0.5%) / 2 (0.5%) | 7 (1.4%) / 6 (1.2%) | 17 (3.6%) / 18 (3.7%) | NA | NA | NA | NA |
| Hao et al. 2022 | NA | 1 (1.47%) / 1 (1.64%) | 3 (4.41%) / 4 (6.56%) | NA | 1 (1.47%) / 1 (1.64%) | NA | NA | NA | NA |
| Tan et al. 2023 HUA TUO | NA | NA | 0 (0.0%) / 0 (0.0%) | NA | NA | NA | 13 (8.2%) / 7 (8.5%) | NA | NA |
| Nicholls et al. 2022 HUYGENS | 0 (0.0%) / 1 (1.2%) | 5 (6.3%) / 6 (7.4%) | NA | NA | NA | NA | NA | NA | NA |
| Janik et al. 2021 | 33 (3.0%) / 12 (1.1%) | NA | 35 (3.2%) / 35 (3.2%) | 14 (1.3%) / 18 (1.7%) | 49 (9.0%) / 50 (8.8%) | 82 (7.5%) / 89 (8.2%) | NA | 61 (5.6%) / 47 (4.3%) | 71 (6.5%) / 85 (7.8%) |
| Ji et al. 2023 | NA | 0 (0.0%) / 4 (7.3%) | 1 (1.8%) / 2 (3.6%) | NA | NA | NA | 1 (1.8%) / 1 (1.8%) | NA | NA |
| Robinson et al.  2014 LAPLACE-2 | 15 (1.3%) / 8 (1.4%) | 17 (1.5%) / 6 (1.1%) | 4 (0.4%) / 6 (1.1%) | 1 (0.1%) / 0 (0%) | NA | NA | NA | NA | 19 (1.7%) / 15 (2.7%) |
| Giugliano 2012 LAPLACE-TIMI 57 | 8 (1.7%) / 3 (1.9%) | NA | 0 (0.0%) / 1 (0.6%) | NA | NA | 48 (10%) / 11 (7%) | NA | NA | NA |
| Lin 2025 NCT05325203 | 6 (6.7%) / 1 (2.2%) | NA | 5 (5.6%) / 2 (4.4%) | NA | NA | NA | 14 (15.6%) / 9 (20.0%) | 9 (10.0%) / 5 (11.1%) | NA |
| McKenney 2012 NCT01288443 | 8 (5.3%) / 0 (0.0%) | 7 (4.6%) / 1 (3.2%) | 0 (0.0%) / 0 (0.0%) | NA | NA | 8 (5.3%) / 1 (3.2%) | 4 (2.6%) / 1 (3.2%) | NA | 6 (4.0%) / 1 (3.2%) |
| Koren 2014 MENDEL-2 | 16 (5.2%) / 8 (5.2%) | 8 (2.6%) / 6 (3.9%) | 3 (1.0%) / 5 (3.2%) | 0 (0.0%) / 0 (0.0%) | NA | 6 (2.0%) / 3 (1.9%) | NA | NA | 10 (3.3%) / 4 (2.6%) |
| Schwartz  2018 ODYSSEY OUTCOMES | 360 (3.8%) / 203 (2.1%) | NA | 500 (5.3%) / 534 (5.7%) | 143 (1.5%) / 167 (1.8%) | 1154 (12.2%) / 1259 (13.3%) | NA | NA | NA | NA |
| Stroes 2016 ODYSSEY CHOICE II | 12 (6.9%) / 0 (0.0%) | 10 (5.8%) / 3 (5.2%) | 1 (0.6%) / 0 (0.0%) | 2 (1.2%) / 0 (0.0%) | NA | 15 (8.7%) / 3 (5.2%) | 7 (4.0%) / 4 (6.9%) | 8 (4.6%) / 1 (1.7%) | 15 (8.7%) / 3 (5.2%) |
| Roth 2016  ODYSSEY CHOICE I | 87 (15.5%) / 18 (8.0%) | 19 (3.4%) / 8 (3.6%) | 6 (1.1%) / 2 (0.9%) | 7 (1.2%) / 2 (0.9%) | 12 (2.1%) / 2 (0.9%) | 49 (8.6%) / 18 (7.9%) | 49 (8.6%) / 18 (7.9%) | 35 (6.1%) / 10 (4.4%) | 35 (6.1%) / 13 (5.7%) |
| Kereiakes 2015 ODYSSEY COMBO I | 11 (5.3%) / 3 (2.8%) | 7 (3.4%)/4 (3.7%) | NA | 0 (0.0%) / 1 (0.9%) | NA | 15 (7.2%) / 5 (4.7%) | 16 (7.7%) / 11 (10.3%) | 13 (6.3%) / 4 (3.7%) | 7 (3.4%)/3 (2.8%) |
| Leiter 2017 ODYSSEY DM-INSULIN | 7 (2.0%)/8 (4.7%) | 15 (4.4%) / 3 (1.8%) | 2 (0.6%)/1 (0.6%) | 4 (1.2%)/0 (0.0%) | 3 (0.9%) / 4 (2.4%) | 17 (4.9%) / 9 (5.3%) | 3 (0.9%) / 4 (2.4%) | 15 (4.4%) / 7 (4.1%) | 10 (2.9%) / 4 (2.4%) |
| Moriarty 2016 ODYSSEY ESCAPE | 1 (2.4%) / 0 (0.0%) | 4 (9.8%) / 1 (4.8%) | 0 (0.0%) / 0 (0.0%) | NA | NA | 4 (9.8%) / 2 (9.5%) | 3 (7.3%) / 4 (19.0%) | NA | 3 (7.3%) / 1 (4.8%) |
| Kastelein 2015 ODYSSEY FH I | 40 (12.4%) / 18 (11.0%) | 6 (1.9%) / 11 (6.7%) | 5 (1.6%) / 2 (1.2%) | 2 (0.6%) / 2 (1.2%) | 6 (1.9%) / 4 (2.5%) | 36 (11.2%) / 12 (7.4%) | 22 (6.8%) / 14 (8.6%) | 13 (4.0%) / 5 (3.1%) | 15 (4.7%) / 9 (5.5%) |
| Kastelein 2015 ODYSSEY FH II | 19 (11.4%) / 6 (7.4%) | 10 (6.0%) / 5 (6.2%) | 6 (3.6%) / 1 (1.2%) | 0 (0.0%) / 1 (1.2%) | 4 (2.4%) / 2 (2.5%) | 21 (12.6%) / 18 (22.2%) | 5 (3.0%) / 1 (1.2%) | NA | 16 (9.6%) / 7 (8.6%) |
| Ginsberg 2016 ODYSSEY HIGH FH | 6 (8.3%) / 2 (5.7%) | NA | 3 (4.2%) / 1 (2.9%) | 1 (1.4%) / 1 (2.9%) | 1 (1.4%) / 1 (2.9%) | NA | NA | NA | NA |
| Blom 2020 ODYSSEY HoFH | 1 (2.2%) / 0 (0.0%) | NA | 0 (0.0%) / 0 (0.0%) | 0 (0.0%) / 0 (0.0%) | 0 (0.0%) / 0 (0.0%) | NA | 2 (4.4%) / 2 (8.3%) | NA | 2 (4.4%) / 2 (8.3%) |
| Teramoto 2016 ODYSSEY JAPAN | 18 (12.6%) / 3 (4.2%) | 2 (1.4%) / 3 (4.2%) | 5 (3.5%) / 1 (1.4%) | NA | NA | 65 (45.5%) / 26 (36.1%) | NA | NA | 5 (3.5%) / 2 (2.8%) |
| Koh 2018 ODYSSEY KT | 2 (2.1%) / 3 (2.9%) | NA | 3 (3.1%)/5 (4.9%) | 0 (0%)/1 (1.0%) | 4 (6.2%)/2 (3.1%) | 6 (6.2%) / 4 (3.9%) | 3 (3.1%) / 6 (5.9%) | NA | 5 (5.2%) / 3 (2.9%) |
| Robinson 2015 ODYSSEY LONG TERM | 91 (5.9%) / 33 (4.2%) | 84 (5.4%) / 23 (2.9%) | 28 (1.8%)/ 18 (2.3%) | 18 (1.2%) / 4 (0.5%) | NA | NA | NA | NA | NA |
| Teramoto 2019 ODYSSEY NIPPON | NA | NA | 0 (0.0%) / 0 (0.0%) | NA | NA | NA | 16 (15.0%) / 9 (16.1%) | NA | NA |
| Bays 2015 ODYSSEY OPTIONS I | 3 (2.9%) / 3 (2.0%) | NA | 0 (0%) / 1 (0.7%) | 0 (0%) / 0 (0%) | NA | 5 (4.8%) / 8 (5.4%) | 5 (4.8%) / (4.7%) | 3 (2.9%) / 8 (5.4%) | NA |
| Farnier 2016 ODYSSEY OPTIONS II | 4 (3.9%) / 2 (2.0%) | NA | 1 (1.0%) / 0 (0%) | 1 (1.0%) / 1 (1.0%) | NA | 4 (3.9%) / 7 (6.9%) | 6 (5.8%) / 9 (8.9%) | NA | NA |
| Ako J 2019 ODYSSEY J-IVUS | 7 (6.8%) / 0 (0%) | NA | NA | NA | 0 (0%) / 3 (2.9%) | 27 (26.2%) / 15 (14.7%) | NA | NA | 4 (3.9%) / 3 (2.9%) |
| Ray  2017  ORION-1 | 19 (5%) / 0 (0%) | 27 (7%) / 6 (5%) | 3 (1%) / 1 (1%) | NA | NA | NA | NA | NA | NA |
| Raal 2024 ORION-5 | 0 (0%) / 0 (0%) | NA | 0 (0%) / 1 (5.3%) | NA | NA | NA | 2 (5.4%) / 2 (10.5%) | NA | NA |
| Raal 2020 ORION-9 | 41 (17.0%) / 4 (1.7%) | NA | 3 (1.2%) / 1 (0.4%) | NA | NA | 28 (11.6%) / 20 (8.3%) | 16 (6.6%) / 16 (6.7%) | NA | NA |
| Ray 2020 ORION-10 | 20 (2.6%) / 7 (0.9%) | NA | 4 (0.5%) / 5 (0.6%) | NA | 120 (15.4%) / 108 (13.9%) | NA | 39 (5.0%) / 33 (4.2%) | NA | NA |
| Ray 2020 ORION-11 | 38 (4.7%) / 4 (0.5%) | NA | 4 (0.5%) / 4 (0.5%) | NA | 88 (10.9%) / 94 (11.7%) | 91 (11.2%) / 90 (11.2%) | 52 (6.4%) / 49 (6.1%) | NA | NA |
| Yamashita 2024 ORION-15 | 21 (8.2%) / 2 (3.5%) | 1 (0.4%) / 0 (0%) | 16 (6.3%) / 2 (3.5%) | NA | 77 (30.2%) / 15 (26.3%) | NA | NA | NA | 1 (0.4%) / 1 (1.8%) |
| Huo 2024 ORION-18 | 5 (2.9%) / 1 (0.6%) | 7 (4.1%) / 1 (0.6%) | 6 (3.5%) / 4 (2.3%) | NA | 15 (8.8%) / 16 (9.2%) | 4 (2.4%) / 2 (1.1%) | 11 (6.5%) / 9 (5.2%) | 10 (5.9%) / 4 (2.3%) | NA |
| Räber 2022 PACMAN-AMI | 9 (6.1%) / 5 (3.3%) | NA | 1 (0.7%) / 0 (0%) | 3 (2.0%) / 0 (0%) | NA | NA | NA | NA | NA |
| Xu 2024 REMAIN-1 | 14 (3.0%) / 4 (1.7%) | NA | 4 (0.9%) / 2 (0.4%) | NA | NA | NA | 8 (1.7%)/ 5 (2.1%) | 3 (0.6%) / 0 (0%) | NA |
| Sun 2024 REMAIN-2 | 18 (3.9%) / 3 (1.3%) | NA | 32 (7.0%) / 9 (3.9%) | NA | 6 (1.3%) / 0 (0%) | NA | 76 (16.7%) / 45 (19.3%) | 43 (9.4%) / 21 (9.0%) | NA |
| Li 2025 REMAIN-3 | 8 (8.4%) / 0 (0%) | 1 (1.1%) / 0 (0%) | 3 (3.2%) / 3 (6.3%) | NA | 1 (1.1%) / 0 (0%) | 0 (0%) / 1 (2.1%) | 1 (1.1%) / 0 (0%) | 1 (1.1%) / 0 (0%) | NA |
| Roth 2012 NCT01288469 | 1 (1.6%) / 2 (6.5%) | NA | 1 (1.6%) / 0 (0%) | NA | NA | NA | NA | NA | 3 (4.9%) / 2 (6.5%) |
| Raal 2012 RUTHERFORD | 7 (6.3%) / 1 (1.8%) | NA | 2 (1.8%) / 0 (0%) | NA | NA | 14 (12.6%) / 6 (10.7%) | 5 (4.5%) / 0 (0%) | NA | 6 (5.4%) / 5 (8.9%) |
| Raal 2014 RUTHERFORD-2 | 13 (5.9%) / 4 (3.7%) | 10 (4.5%) / 1 (0.9%) | 0 (0%) / 0 (0%) | 0 (0%) / 0 (0%) | NA | 19 (8.6%) / 5 (4.6%) | NA | NA | 9 (4.1%) / 4 (3.7%) |
| Shao 2025 NCT05621070 | 5 (5.4%) / 0 (0%) | NA | 2 (2.2%) / 8 (17.0%) | NA | NA | NA | 5 (5.4%) / 2 (4.3%) | 9 (9.8%) / 2 (4.3%) | NA |
| Stein 2012 NCT01266876 | 25 (40.3%) / 2 (13.3%) | NA | 0 (0%) / 0 (0%) | NA | NA | NA | NA | NA | NA |
| Uehara 2024 Adage-Joto | NA | NA | NA | NA | NA | NA | NA | NA | NA |
| Wang Zhao 2024 | 1 (0.6%) / 0 (0%) | NA | 8 (4.7%) / 2 (2.3%) | NA | 6 (3.6%) / 2 (2.3%) | NA | NA | 7 (4.1%) / 6 (7.0%) | NA |
| Wang 2024 NCT04781114 | 18 (3.4%) / 4 (1.5%) | NA | 40 (7.4%) / 20 (7.5%) | NA | 23 (4.3%) / 19 (7.2%) | NA | 58 (10.8%) / 35 (13.2%) | 53 (9.9%) / 31 (11.7%) | NA |
| Hirayama 2014 YUKAWA | 4 (2.0%) / 1 (1.0%) | 4 (2.0%) / 1 (1.0%) | 1 (0.5%) / 0 (0%) | NA | NA | NA | NA | NA | NA |
| Kiyosue  2016 YUKAWA-2 | 5 (2.5%) / 5 (2.5%) | 1 (0.5%) / 2 (1.0%) | 1 (0.5%) / 2 (1.0%) | 0 (0%) / 0 (0%) | 4 (2.0%) / 4 (2.0%) | 34 (16.8%) / 36 (17.8%) | NA | NA | NA |
| Koren 2012 MENDEL | 15 (6%) / 4 (4%) | 7 (2.6%) / 0 (0%) | 2 (0.7%) / 0 (0%) | NA | NA | 11 (4.1%) / 3 (3.3%) | 17 (6.3%) / 7 (7.8%) | NA | 8 (3.0%) / 4 (4.4%) |
| Teramoto 2016 NCT01812707 | 7 (9.3%) / 1 (4.0%) | NA | 1 (1.3%) / 0 (0%) | NA | NA | 16 (21.3%) / 2 (8.0%) | NA | NA | NA |

Data are presented as the number and/or percentage of participants with each adverse event, as reported in the original studies. NA indicates that the corresponding adverse event was not reported in the original publication and should not be interpreted as indicating that no event occurred. For muscle-related events, the event category reported by the original study was extracted directly when available; when no overall muscle-related event category was provided, myalgia was extracted as the representative event in most studies. Hepatic effects included reported elevations or abnormalities in alanine aminotransferase (ALT) and/or aspartate aminotransferase (AST). Definitions and laboratory thresholds for ALT/AST abnormalities were retained as reported in the original studies and may therefore differ across trials.
